# Genome-wide association studies of missing metabolite measures from two population-based studies

**DOI:** 10.1186/s13059-026-04132-9

**Published:** 2026-07-10

**Authors:** Tariq O. Faquih, Mohammed Aslam Imtiaz, Valentina Talevi, Elvire N. Landstra, Astrid van Hylckama Vlieg, Ruifang Li-Gao, Frits R. Rosendaal, Raymond Noordam, Diana van Heemst, Dennis O. Mook-Kanamori, Monique M. B. Breteler, N. Ahmad Aziz, Ko Willems van Dijk

**Affiliations:** 1 Department of Clinical Epidemiology, Leiden University Medical Center, Leiden, The Netherlands; 2 Division of Sleep and Circadian Disorders, Brigham and Women’s Hospital, Boston, USA; 3 King Faisal Specialist Hospital & Research Center, Riyadh, Kingdom of Saudi Arabia; 4 German Centre for Neurodegenerative Diseases (DZNE), Population Health Sciences, Bonn, Germany; 5 Department of Internal Medicine, Section of Gerontology and Geriatrics, Leiden University Medical Center, Leiden, The Netherlands; 6 Department of Public Health and Primary Care, Leiden University Medical Center, Leiden, The Netherlands; 7 University of Bonn, University Hospital Bonn, Institute for Medical Biometry, Informatics and Epidemiology (IMBIE), Faculty of Medicine, Bonn, Germany; 8 Center for Neurology, University of Bonn, University Hospital Bonn, Bonn, Germany; 9 Department of Human Genetics, Leiden University Medical Center, Leiden, The Netherlands; 10 Department of Internal Medicine, Division of Endocrinology, Leiden University Medical Center, Leiden, The Netherlands; 11 Einthoven Laboratory for Experimental Vascular Medicine, Leiden University Medical Center, Leiden, The Netherlands

**Keywords:** Genome-wide association study, Missing measurements, Inborn errors of metabolism, Metabolomics, Poor metabolizers

## Abstract

**Background:**

Metabolomic analyses are increasingly applied in both etiological and predictive research, but frequently report missing values, which are then either imputed or removed from the analyses, and not examined as true missingness due to altered metabolism. We hypothesized that interindividual genetic variation may account for part of this missingness.

**Results:**

We perform a logistic GWAS of metabolite missingness from an untargeted mass spectrometry-based platform in the Netherlands Epidemiology of Obesity Study (*N* = 594) and the Rhineland Study (*N* = 4,165). We consider metabolites missing in 10%-90% of individuals in both cohorts (*N* = 224). GWAS meta-analyses of these metabolites’ probability of missingness revealed 55 metabolome-wide significant associations, including 42 novel ones (*p* < 1.58 × 10^–10^), involving 28 metabolites and 41 lead SNPs.

**Conclusions:**

Despite considerable pleiotropy, the majority of identified SNP- ‘missing metabolite’ associations are biologically plausible, relating to beta-oxidation, bile acids, steroids, and xenobiotics metabolism. These findings suggest that missing values in metabolomics are partially non-random and reflect potential genetic variation.

**Supplementary Information:**

The online version contains supplementary material available at 10.1186/s13059-026-04132-9.

## Background

Metabolites are small molecules that are produced or consumed during anabolic or catabolic reactions and constitute the basic building blocks of all biological processes. Circulating metabolite levels are thought to reflect the integrated metabolic response to changes in genetic and non-genetic (including dietary and other environmental) factors [1]. This hypothesis has made metabolomics an attractive field of study for elucidating the biological mechanisms underlying complex multifactorial diseases [1, 2]. Recent advances in metabolomics have enabled high-throughput analysis of thousands of metabolites from a single biological sample, and have been applied to study a wide range of cardiovascular [3, 4], metabolic [5, 6], and neurodegenerative outcomes [7, 8], as well as other traits [9–11].

The field of metabolomics remains relatively new and still faces several challenges. One important challenge is the biological meaning of missing measurements of metabolites, particularly with untargeted approaches that are designed to maximize the number of measured metabolites at the cost of lower specificity [12, 13]. Conceptually, missing data could be due to either random or systematic (i.e., technical) measurement errors, or reflect the actual absence of specific metabolites. In addition, when the metabolite concentration in the sample is below the limit of detection of the measurement method, it will be reported as a missing value [12, 13]. Indeed, in most studies, missing data are assumed to reflect values below the limit of detection, and consequently are either removed from the analysis or imputed [12, 13]. However, a priori, it cannot be ruled out that missing values of metabolites are caused by genetic variations. In this case, the metabolites with missing values could be truly absent from the sample due to functional alterations of specific biological pathways driven by genetic variation [9, 14, 15]. Therefore, imputation or removal of those metabolites from the analysis could bias biological interpretation.

Long before large-scale metabolomics data became available, rare genetic mutations affecting metabolism were identified and investigated [16]. Disorders caused by genetic mutations that disrupt metabolism are referred to as inborn errors of metabolism (IEM). Usually, the causal genetic mutations are located in protein coding genes and affect the structure of the encoded proteins to such an extent that their biological function is disrupted [17]. For example, IEM disorders can disrupt carbohydrate metabolism, protein metabolism, fatty acid oxidation, and glycogen storage [17, 18]. Collectively, IEM disorders have an overall incidence of 1 in 2,500 births [18]. IEM illustrate that certain genetic variants have the potential to prevent the synthesis or breakdown of specific metabolites by disrupting metabolic pathways [19].

We set out to test the hypothesis that at least some of the common missing values in metabolomics data, either due to levels below the limit of detection or otherwise, are caused by common genetic variation. We also hypothesized that the nature and context of the potential associations could provide insights into the potential causes of the missingness (i.e., technical, below the limit of detection, or truly absent). To address these hypotheses, we performed genome-wide association studies (GWAS) to discover SNPs associated with the probability of absence (i.e., ‘missingness’ due to concentrations below the limit of detection, technical errors, or truly absent) of metabolite measures.

## Results

### Discovery genome-wide association studies of missing metabolites

**Fig. 1:**
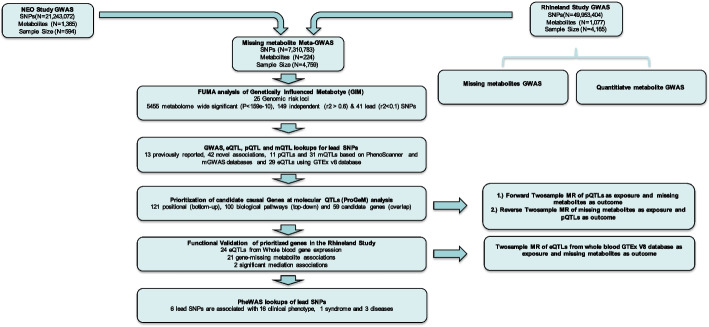
Workflow of the GWAS and post-GWAS analyses of missing metabolite measures

The GWAS of missing metabolite measures was performed separately in 594 individuals from the NEO study (mean (standard deviation (SD)) age: 55.8 (5.9), range: 45–66 years, 53% women), and 4,165 individuals from the Rhineland Study (mean (SD) age: 55.5 (14), range: 30–96 years, 56% women). Individual study characteristics and general genotype assay information are summarized in Table 1. GWAS results in NEO identified 712 metabolome-wide significant (*p* < 1.58 × 10^–10^) associations between 537 SNPs and 6 out of the 341 included metabolites. In the Rhineland Study, we identified 4,370 metabolome-wide significant (*p* < 1.59 × 10^–10^) associations between 2,615 SNPs and 32 out of the 425 included metabolites. Restricted to the metabolites that were available in both studies (*N* = 224), the study-specific GWAS identified 523 and 2,613 metabolome-wide significant SNPs for 5 and 26 metabolites in the NEO and the Rhineland study, respectively. The overall workflow of the GWAS analysis and following downstream analyses is illustrated in Fig. 1. The summary statistics of GWAS analysis are available in Additional file 2: Table S1 and Additional file 3: Table S2 for NEO and the Rhineland Study, respectively. In the sensitivity analysis adjusting for prevalent diabetes in the Rhineland Study, the main findings remained largely unchanged. However, genomic risk loci 2 (*ALMS1*) and 12 (*PYROXD2*) that were associated with N2-acetyl, N6, N6-dimethyllysine, and genomic risk locus 20 (*CRX*) that was associated with tetrahydrocortisol sulfate were no longer statistically significanct. Among these, only N2-acetyl, N6, N6-dimethyllysine showed directional inconsistency in SNP effect size estimates between the primary model and the sensitivity analysis (Additional file 3: Table S2).

**Table 1:** Overview of the sample characteristics and general genotype assay information

Characteristic	NEO study (*n* = 594)	Rhineland study (*n* = 4165)
Age: mean (SD)	55.8 (5.9)	55.5 (13.96)
Sex: % female	53%	56%
Metabolomics platform	Metabolon-HD4	Metabolon-HD4
N of metabolites with missing values	433	340
Blood sampling	Serum	Plasma
Fasting status %	100%	99.4%
Genotyping Array	Illumina HumanCoreExome-24 BeadChip	Omni 2.5 Exome Array
Genotype Imputation Panel	HRC (r1.1)	1000 genome phase 3 version 5

*SD *Standard deviation

### Quantitative GWAS sensitivity analysis

For the metabolome-wide significant hits identified in missing metabolites, we assessed whether the associated loci had been replicated in quantitative GWAS. Seventeen out of twenty-six metabolites exhibited unique loci in the missing metabolite GWAS that were not observed in their corresponding quantitative GWAS. These metabolites included 3-decenoylcarnitine, 5alpha-androstan-3alpha,17alpha-diol monosulfate, 5alpha-pregnan-diol disulfate, X-13658, X-12753, X-12410, alliin, dopamine 4-sulfate, estrone 3-sulfate, ferulic acid 4-sulfate, glycocholate glucuronide (1), N2-acetyl,N6,N6-dimethyllysine, N-acetylkynurenine (2), pregnanolone/allopregnanolone sulfate, and tetrahydrocortisol sulfate (Additional file 1: Fig. S1, Additional file 3: Table S2).

### Study-specific SNP x SNP interaction

For all 224 metabolites overlapping in both NEO and the Rhineland study, we did not observe any metabolome-wide significant SNP × SNP interactions across the studies, except for 22 SNPs associated with 10 metabolites that reached genome-wide significance (Additional file 4: Table S3). Our results indicated that for the majority of SNPs among overlapping metabolites, no SNP × SNP interactions were detected between the NEO and the Rhineland Study.

### Genome-wide meta-analysis of missing metabolites

**Fig. 2:**
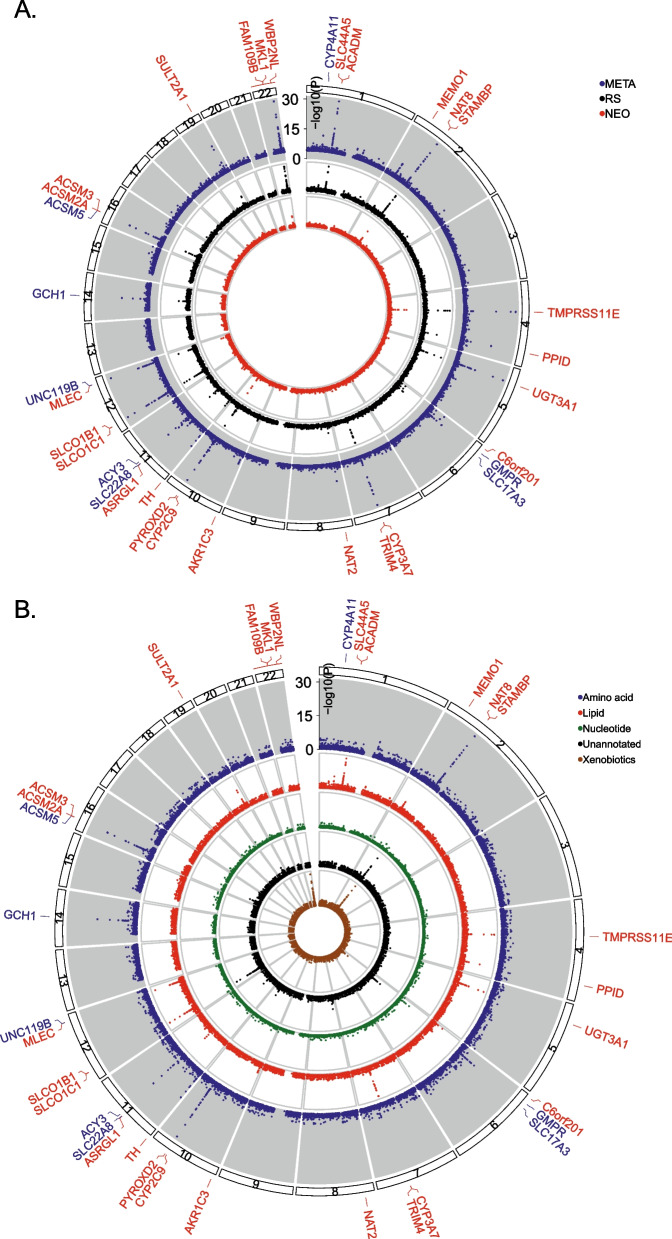
Genomic loci associated with missingness of metabolites. **A** Circular Manhattan plot showing both cohort-specific and meta-analysis GWAS results. **B** Circular Manhattan plot showing regional associations of genomic locations per metabolite class. The color of the genes indicates whether the identified lead SNP locus is novel. Dark blue indicates lead SNP loci (r2 < 0.1) that were previously reported, and red lead SNP loci that were identified to be novel with metabolites. The *p*-value axis is truncated at *p* < 1 × 10^–30^ for visualization purposes. All GWAS models were adjusted for age, sex, batch, fasting status and population substructure principal components

The meta-analysis of the overlapping 224 metabolites in the two studies identified 5,455 significant associations (*p* < 1.59 × 10^–10^), including 3,260 SNPs across 33 different metabolites (Fig. 2 and Additional file 6: Table S5). The frequencies of missingness across the 224 metabolites in the NEO and Rhineland Study cohorts are provided in Additional file 5: Table S4. The direction of the associations was similar across both cohorts (Pearson correlation R2 = 0.92) using independent SNP-metabolite (r^2^ < 0.6) associations (Additional file 1: Fig. S2 and Additional file 7: Table S6). The majority of these metabolites belonged to the steroid metabolism pathway (*N* = 7), followed by amino-acid metabolism (*N* = 4), fatty acid metabolism (*N* = 4), bile acid metabolism (*N* = 5), and unannotated metabolites (*N* = 8). Other hits belonged to food and plant-derived xenobiotics (i.e., alliin, solanidine, ferulic acid 4-sulfate, and caffeic acid sulfate) and nucleotide metabolites (xanthosine).

### Genetically influenced metabotypes

**Fig. 3:**
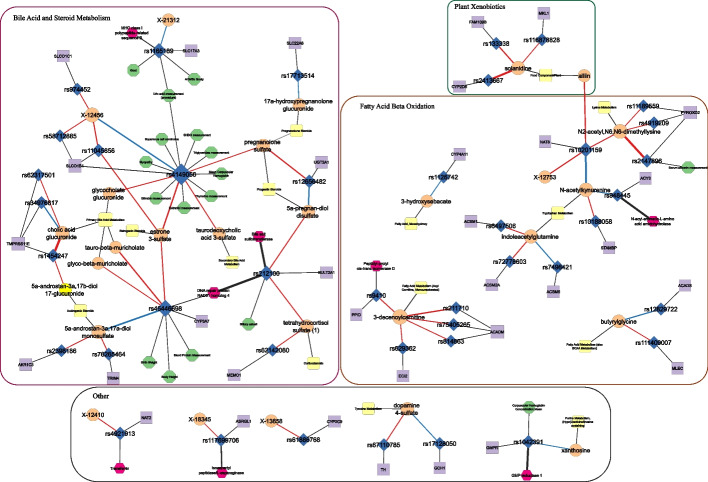
Network representation of the 41 lead SNP-metabolite associations. The network shows the associations between the 41 lead SNPs (blue diamonds) and metabolites (orange circles). The network also includes the mapped genes to each SNP (purple squares), assigned sub-pathway from the measurement platform (yellow rounded squares), traits and diseases associated with the SNPs from DisGeNET (green octagons), and pQTL associations from Phenoscanner (pink hexagons). Novel and reported associations between the SNP-metabolites are represented in red- and orange-colored lines respectively

Genetically Influenced Metabotypes (GIMs), defined as variant-metabolite clusters [9], were identified by merging the summary statistics of all SNP-metabolite associations and selecting the SNPs with the lowest association *P*-value, resulting in a set of 7,310,783 unique SNPs. Functional Mapping and Annotation (FUMA) identified 3,260 metabolome-wide significant SNPs indexed by 41 lead SNPs (linkage disequilibrium (LD) r^2^ < 0.1) located across 25 genomic risk loci (Additional file 7: Table S6 and Additional file 8: Table S7). Those 41 lead SNPs had a total of 55 associations with the odds of missingness of 28 metabolites. Those 55 lead SNP-metabolite associations were further cross-referenced with previous metabolomic GWAS results and metabolome-based GWAS databases to assess the novelty of the associations (Table 2 and Additional file 9: Table S8). We defined SNP-metabolite associations as “novel” if the SNP-metabolite association was not reporte'd previously, and labelled it as “reported” otherwise. Accordingly, we found 42 novel and 13 previously reported associations. Several of the associated metabolites were connected by shared pathways and genes, as shown in Fig. 3, and formed two large clusters. The first cluster was enriched for steroids and bile acid metabolites and contained two pleiotropic SNPs (rs4149056 and rs45446698) associated with 5 and 4 metabolites, respectively. The second cluster comprised acetylated tryptophan and lysine related metabolites, along with the xenobiotic metabolite alliin. An interactive version of this network is available online at https://tofaquih.github.io/GWASMissingMetabolites/ for further exploration.

**Table 2:** Novel and previously reported lead SNP-Metabolite associations using PhenoScanner

Sub pathway	Metabolite name	Missing frequency NEO/RS	SNP	nearestGene	Beta (SE)	*P* value	Novelty	HMDB
Androgenic Steroids	5alpha-androstan-3alpha,17alpha-diol monosulfate		rs2398186	*AKR1C3*	0.4495 (0.0684)	5.004 × 10^–11^	Novel	HMDB0000458
		23.4/27.4	rs45446698	*CYP3A7*	−2.3785 (0.1532)	2.441 × 10^–54^	Reported	
			rs76265464	*TRIM4*	−2.014 (0.249)	6.07 × 10^–16^	Novel	
	5alpha-androstan-3alpha,17beta-diol 17-glucuronide	55.72/55.75	rs1454247	*TMPRSS11E*	−0.5275 (0.0717)	1.931 × 10^–13^	Novel	
Corticosteroids	tetrahydrocortisol sulfate (1)	57.74/48.49	rs212100	*SULT2A1*	0.7805 (0.0898)	3.556 × 10^–18^	Novel	HMDB0000949
			rs62142080	*MEMO1*	0.4637 (0.0657)	1.636 × 10^–12^	Novel	
Estrogenic Steroids	estrone 3-sulfate	79.46/61.56	rs11045856	*SLCO1B1*	−0.5496 (0.0584)	4.551 × 10^–21^	Novel	HMDB01425
			rs4149056	*SLCO1B1*	0.8336 (0.0634)	1.853 × 10^–39^	Novel	
			rs45446698	*CYP3A7*	−1.9913 (0.2056)	3.486 × 10^–22^	Novel	
Fatty Acid Metabolism (Acyl Carnitine, Monounsaturated)	3-decenoylcarnitine	19.53/29.2	rs211710	*SLC44A5*	−0.7772 (0.0612)	5.372 × 10^–37^	Novel	HMDB0241067
			rs629362	*C6orf201*	0.4453 (0.0597)	8.92 × 10^–14^	Novel	
			rs75405265	*ACADM*	−1.3468 (0.1613)	6.778 × 10^–17^	Novel	
			rs814863	*SLC44A5*	−0.617 (0.0883)	2.75 × 10^–12^	Novel	
			rs9410	*PPID*	0.5561 (0.0604)	3.23 × 10^–20^	Novel	
Fatty Acid Metabolism (also BCAA Metabolism)	butyrylglycine	45.96/61.56	rs111409007	*MLEC*	0.4744 (0.0722)	5.008 × 10^–11^	Novel	HMDB00808
			rs12829722	*UNC119B*	0.6893 (0.0605)	4.881 × 10^–30^	Reported	
Fatty Acid, Monohydroxy	3-hydroxysebacate	10.77/17.14	rs1126742	*CYP4A11*	−0.5556 (0.078)	1.085 × 10^–12^	Reported	HMDB0340579
Food Component/Plant	alliin	67.68/60.48	rs10201159	*NAT8*	−0.7113 (0.0588)	1.075 × 10^–33^	Novel	HMDB33592
	solanidine	80.13/73.88	rs116878828	*MKL1*	0.582 (0.0893)	7.267 × 10^–11^	Novel	HMDB03236
			rs133338	*WBP2NL*	0.7751 (0.0648)	5.661 × 10^–33^	Novel	
			rs2413667	*FAM109B*	1.1182 (0.0608)	2.024 × 10^–75^	Novel	
Lysine Metabolism	N2-acetyl,N6,N6-dimethyllysine	16.67/13.28	rs10201159	*NAT8*	0.8927 (0.0986)	1.334 × 10^–19^	Novel	
			rs11189559	*PYROXD2*	0.4777 (0.0743)	1.317 × 10^–10^	Novel	
			rs2147896	*PYROXD2*	−1.2699 (0.0649)	3.051 × 10^–85^	Novel	
			rs4919209	*PYROXD2*	0.4708 (0.0724)	8.003 × 10^–11^	Novel	
Pregnenolone Steroids	17alpha-hydroxypregnanolone glucuronide	44.78/64.64	rs17713514	*SLC22A8*	1.5434 (0.2094)	1.717 × 10^–13^	Reported	HMDB0000363
Primary Bile Acid Metabolism	cholic acid glucuronide	39.56/39.88	rs1454247	*TMPRSS11E*	−1.038 (0.0502)	8.29 × 10^–95^	Novel	HMDB0002577
			rs34976817	*TMPRSS11E*	−0.9448 (0.0802)	4.947 × 10^–32^	Reported	
			rs62317501	*TMPRSS11E*	−0.4206 (0.0638)	4.458 × 10^–11^	Novel	
	glyco-beta-muricholate	29.12/16.57	rs45446698	*CYP3A7*	1.8743 (0.2818)	2.888 × 10^–11^	Novel	HMDB0341323
	glycocholate glucuronide	79.63/66.75	rs4149056	*SLCO1B1*	0.4621 (0.0607)	2.718 × 10^–14^	Novel	HMDB0341324
	tauro-beta-muricholate	70.03/51.81	rs45446698	*CYP3A7*	2.0306 (0.1632)	1.526 × 10^–35^	Novel	HMDB0000932
Progestin Steroids	5alpha-pregnan-diol disulfate	56.06/40.89	rs12656482	*UGT3A1*	1.0108 (0.0788)	1.052 × 10^–37^	Reported	
			rs212100	*SULT2A1*	0.6352 (0.0697)	8.317 × 10^–20^	Novel	
	pregnanolone/allopregnanolone sulfate	53.2/47.54	rs12656482	*UGT3A1*	0.8004 (0.0678)	3.371 × 10^–32^	Novel	HMDB0062782
			rs4149056	*SLCO1B1*	0.4155 (0.0618)	1.834 × 10^–11^	Novel	
Purine Metabolism	xanthosine	23.4/59.98	rs1042391	*GMPR*	0.3338 (0.0467)	8.819 × 10^–13^	Reported	HMDB0000299
Secondary Bile Acid Metabolism	taurodeoxycholic acid 3-sulfate	30.81/21.37	rs4149056	*SLCO1B1*	0.6427 (0.0784)	2.525 × 10^–16^	Novel	HMDB0240734
Tryptophan Metabolism	indoleacetylglutamine	25.42/14.07	rs6497506	*ACSM3*	0.5559 (0.0713)	6.353 × 10^–15^	Novel	HMDB0013240
			rs72778603	*ACSM2A*	−1.2624 (0.1369)	2.882 × 10^–20^	Novel	
			rs7498421	*ACSM5*	0.6891 (0.0686)	9.52 × 10^–24^	Reported	
	N-acetylkynurenine	43.43/33.76	rs10188058	*STAMBP*	0.6201 (0.0793)	5.423 × 10^–15^	Novel	HMDB0240342
			rs10201159	*NAT8*	1.2782 (0.0738)	3.755 × 10^–67^	Reported	
			rs948445	*ACY3*	0.6161 (0.0677)	9.101 × 10^–20^	Reported	
Tyrosine Metabolism	dopamine 4-sulfate	15.32/59.98	rs17128050	*GCH1*	−0.6634 (0.0754)	1.447 × 10^–18^	Reported	HMDB0004148
			rs67110785	*TH*	0.4338 (0.0537)	6.913 × 10^–16^	Novel	
	X-12410	15.66/11.98	rs4921913	*NAT2*	−0.6524 (0.087)	6.57 × 10^–14^	Novel	
	X-12456	56.9/51.98	rs11045856	*SLCO1B1*	−0.5904 (0.0554)	1.635 × 10^–26^	Novel	
			rs4149056	*SLCO1B1*	0.8525 (0.0647)	1.098 × 10^–39^	Reported	
			rs58712885	*SLCO1B1*	−0.6786 (0.1035)	5.548 × 10^–11^	Novel	
			rs974452	*SLCO1C1*	−0.5143 (0.0723)	1.119 × 10^–12^	Novel	
	X-12753	43.94/69.99	rs10201159	*NAT8*	0.5979 (0.055)	1.729 × 10^–27^	Novel	
	X-13658	84.34/50.83	rs61886768	*CYP2C9*	0.4206 (0.0598)	2.066 × 10^–12^	Novel	
	X-18345	59.43/31.69	rs117699706	*ASRGL1*	−0.5411 (0.0802)	1.537 × 10^–11^	Novel	
	X-21312	50.84/29.72	rs1165189	*SLC17A3*	0.477 (0.0604)	2.751 × 10^–15^	Reported

### Identification of eQTLs, pQTLs and mQTLs

Using GTEx (version 8), we identified 29 expression Quantitative Trait Loci (eQTLs) (Additional file 9: Table S8). Interestingly, among the novel associations, the SNP rs2413667, which was associated with the odds of missingness of solanidine, was an eQTL of *CYP2D6*, an important enzyme for xenobiotic metabolism [22], in adipose tissue. The PhenoScanner results showed that five lead SNPs were previously reported as protein Quantitative Trait Loci (pQTLs) for seven different proteins (Table 3 and Additional file 10: Table S9). These included peptidyl-prolyl cis–trans isomerase D, major histocompatibility class I polypeptide-related sequence B, and DNA repair protein RAD51 homolog. Finally, using the metabolome-based GWAS study databases, we identified 27 lead SNPs as metabolome Quantitative Trait Loci (mQTLs) (Additional file 11: Table S10). For example, we identified rs211710, which was associated with the odds of missingness of 3-decenoylcarnitine in our study, as an mQTL for decenoylcarnitine.

**Table 3:** Top pQTL associations with the lead SNPs

Metabolite name	Sub pathway	Lead SNP	Nearest gene	Proxy pQTL SNPs*	pQTL associated trait (Phenoscanner)	pQTL associated trait (UK biobank)
3-decenoylcarnitine	Fatty Acid Metabolism (Acyl Carnitine. Monounsaturated)	rs9410	*PPID*	rs8396	Peptidyl-prolyl cis–trans isomerase D	-
X-21312	-	rs1165189	*SLC17A3*	rs13200784, rs3757132	MHC class I polypeptide-related sequence B	-
estrone 3-sulfate	Estrogenic Steroids	rs45446698	*CYP3A7*	rs45446698	DNA repair protein RAD51 homolog 4	-
tauro-beta-muricholate	Primary Bile Acid Metabolism					-
5a-Androstane-3a,17a-diol monosulfate	Androgenic Steroids					-
glyco-beta-muricholate	Primary Bile Acid Metabolism					-
X-12410	-	rs4921913	*NAT2*	rs4921915	Transferrin	-
5alpha-pregnan-diol disulfate	Progestin Steroids	rs212100 rs212100	*SULT2A1* *SULT2A1*	rs212100 rs212100	Bile salt sulfotransferase,DNA repair protein RAD51 homolog 4 Bile salt sulfotransferase , DNA repair protein RAD51 homolog 4	SULT2A1 SULT2A1
tetrahydrocortisol sulfate	Corticosteroids					
X-18345	-	rs117699706	*ASRGL1*	-	-	Isoaspartyl peptidase/L-asparaginase
N-acetylkynurenine (2)	Amino acid	rs948445	*ACY3*	-	-	N-acyl-aromatic-L-amino acid amidohydrolase (carboxylate-forming)
xanthosine	Nucleotide	rs1042391	*GMPR*	-	-	GMP reductase 1

^*^Proxy SNPs selected based on their correlation (r^2^) with the lead SNP. Full results available in Table S7

### Prioritized candidate genes for missing metabolites

**Fig. 4:**
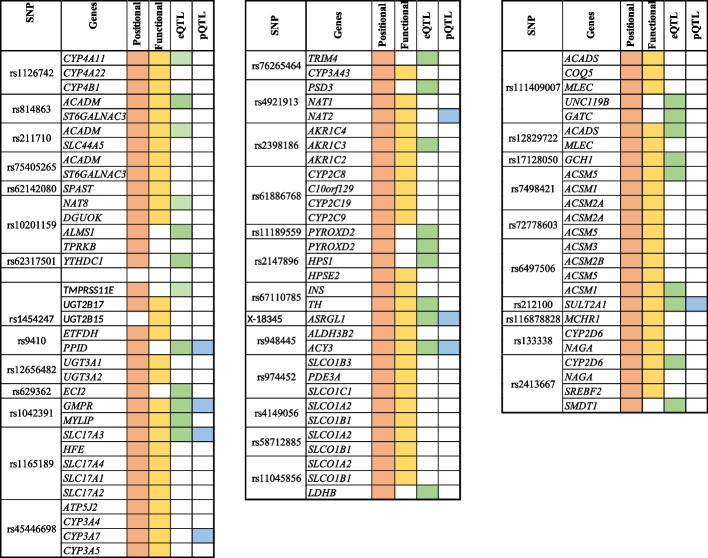
Summary of top ranked candidate gene identification, eQTL, and pQTL associations per identified SNP-metabolite. Candidate genes identified by the ProGeM positional approach (bottom-up) are highlighted in orange under the “positional” column and highlighted in yellow under the “functional” column based on metabolic and phenotypic relevance approach (top-down). Genes with a significant eQTL association are highlighted in green under the eQTL column and in blue for SNP-pQTL associations under the pQTL column

To map the 41 identified lead SNPs associated with the odds of missingness of the metabolites to candidate casual genes, we used the ‘Prioritization of candidate causal Genes at Molecular QTLs’ (ProGeM) framework. This framework maps SNPs to genes using two complementary methods. The first is based on positional proximity, which mapped the identified SNPs to 121 genes. The second is based on biological relevance, which mapped the SNPs to 100 relevant genes (Fig. 4, Additional file 12: Table S11 and Additional file 13: Table S12). Subsequently, we focused the analysis on genes that were mapped through both methods, resulting in 59 candidate causal genes.

### Mediation analysis between SNP, gene expression levels, and odds of missingness

Out of the 162 identified genes, we conducted a functional analysis for 78 genes of which expression levels were available in 2,575 participants of the Rhineland Study. We found that 18 lead SNPs were significantly associated with 20 genes at a nominal p-value threshold—with the relation between rs10201159 and *ALMS1* emerging as the strongest association (β = −1.55, p-value < 0.001) (Additional file 14: Table S13). The expression levels of 18 genes were significantly associated with the odds of missingness for 11 metabolites—with the *HPS1* and N2-acetyl, N6,N6-dimethyllysine association emerging as the strongest (β = −0.45, p-value < 0.001). Finally, the mediation analysis (Table 4) conducted involving 9 genes whose expression levels were significantly associated with both lead SNPs and the corresponding metabolites, indicated that *HPS1* (unadjusted p-value = 0.004, FDR-adjusted p-value = 0.04) significantly partially mediated the effect between the rs2147896 SNP and the odds of missingness of the N2-acetyl,N6.N6-dimethyllysine metabolite by 2.8% (β (SE) = −0.02 (0.007)). This was followed by *SMDT1* (unadjusted p-value = 0.06, FDR p-value = 0.25) that showed suggestive but non-significant partial mediation with the effect between rs2413667 and solanidine by 3.2% (β (SE) = −0.02 (0.01)).

**Table 4:** eQTL mediation of the association between the lead SNPs and missing metabolites by primary candidate genes

**Exposure**	**Mediator**	**Metabolite**	**Indirect effect**			**Direct effect**		**Total effect**		**Percentage of mediation**^**±**^
			**β estimate (SE)**	***P*** value	**FDR** **pval**	**β estimate (SE)**	***P*** value	**β estimate (SE)**	***P*** value	
rs117699706	*ASRGL1*	X-18345	−0.030 (0.023)	0.212	0.382	−0.30 (0.067)	6.6 × 10^–06^	−0.333 (0.062)	9.6 × 10^–08^	8.9%
rs629362	*ECI2*	3-Decenoylcarnitine	−0.015 (0.013)	0.259	0.388	−0.236 (0.047)	7.6 × 10^–07^	−0.252 (0.045)	2.9 × 10^–08^	6.2%
rs10201159	*ALMS1*	X-12753	−0.025 (0.025)	0.320	0.411	−0.407 (0.053)	1.8 × 10^–14^	−0.433 (0.046)	< 10 × 10^–16^	5.9%
rs814863	*ACADM*	3-Decenoylcarnitine	−0.011 (0.007)	0.172	0.382	−0.319 (0.063)	4.9 × 10^–07^	−0.330 (0.063)	2.0 × 10^–07^	3.2%
rs2413667	*SMDT1*	Solanidine	−0.024 (0.012)	0.055	0.247	−0.722 (0.050)	< 10 × 10^–16^	−0.746 (0.047)	< 10 × 10^–16^	3.2%
rs10201159_T	*ALMS1*	N2-acetyl,N6,N6-dimethyllysine	−0.015 (0.035)	0.684	0.731	−0.495 (0.074)	2.9 × 10^–11^	−0.509 (0.066)	1.3 × 10^–14^	2.9%
rs2147896	*HPS1*	N2-acetyl,N6,N6-dimethyllysine	−0.022 (0.007)	0.004	0.036*	−0.745 (0.046)	< 10 × 10^–16^	−0.766 (0.046)	< 10 × 10^–16^	2.8%
rs11189559_G	*PYROXD2*	N2-acetyl,N6,N6-dimethyllysine	−0.005 (0.004)	0.176	0.382	−0.236 (0.049)	1.9 × 10^–06^	−0.241 (0.049)	1.1 × 10^–06^	2.2%
rs211710	*ACADM*	3-Decenoylcarnitine	−0.002 (0.006)	0.731	0.731	−0.556 (0.048)	< 10 × 10^–16^	−0.557 (0.048)	< 10 × 10^–16^	0.4%

^*^Indirect effect’s FDR pval = < 0.05

^± (^Indirect effect/total effect) * 100

### Missing metabolites variants implicated in diseases

Our search in the DisGeNET database, using curated data, revealed that six lead SNPs were related to sixteen phenotypes, three diseases, and one syndrome (Additional file 15: Table S14). The phenotypes were either related to liver, kidney, or reproductive function. Notably, rs4149056 was associated with 7 phenotypes, including levels of thyroxine, sex hormone binding globulin (SHBG), and estradiol. Another notable SNP was rs45446698, which was related to three phenotypes, including birth body weight, body height, and blood protein measurement. Lastly, in the disease category, we found the rs4149056 SNP to be associated with squamous cell carcinoma.

### Mendelian randomization

For the eQTL based MR analysis, we identified two associations based on the IVW: *ACADM* with 3-decenoylcarnitine (IVW: β = 3.12, SE = 0.41, FDR *p*-value = 1.6 × 10^–13^), and *CYP2D6* with solanidine (IVW: β = −0.36, SE = 0.158, FDR *p*-value = 0.03). For the pQTL MR analysis, we identified 5 associations using IVW MR (forward). Notable associations were between RAD51D with 5alpha-androstan-3alpha,17alpha-diol monosulfate (IVW: β = 1.98, SE = 0.23, FDR *p*-value = 5.51 × 10^–16^, MR-radial IVW: β = 2.11, SE = 0.12, *p*-value = 3.88 × 10^–66^)—which had the largest effect and significance—and between ACY3 with N-acetylkynurenine (IVW: β = −0.93, SE = 0.266, FDR *p*-value = 0.001, MR-radial IVW: β = −0.83, SE = 0.24, *p*-value = 0.005). In the reverse pQTL MR analysis, we identified 3 associations. Notable associations were 5alpha-androstan-3alpha,17alpha-diol monosulfate with RAD51D (IVW: β = 0.43, SE = 0.07, FDR *p*-value = 1.49 × 10^–9^; MR-radial IVW: β = 2.1, SE = 0.12, *p*-value = 3.88 × 10^–66^), the only association found in both the forward and reverse MR analyses, and X-12410 with transferrin (IVW: β = −0.11, SE = 0.05, FDR *p*-value = 0.03). All significant associations are presented in Table 5, and the full results of the MR analyses are provided in Additional file 16: Table S15 and Additional file 17: Table S16.

**Table 5:** Inverse variance weighted Two-sample MR associations for pQTLs and eQTLs with missing metabolites

Exposure	Outcome	N SNPs	Beta (CI)	pvalue	FDR pval	F	Q	Q_pval	Beta (CI)	Radial-MR pvalue^±^
ACY3	N-acetylkynurenine	20	−0.93 (−1.45, −0.41)	0.0004	0.00102	118.49	65.6	4.807 × 10^–7^*	−0.745 (−1.064, −0.426)	0.005
GMPR1	xanthosine	5	1.15 (0.59, 1.71)	5.36 × 10^–5^	0.000196	209.9	10.3	0.035*	**-**	**-**
RAD51D	5alpha-androstan-3alpha,17alpha-diol monosulfate	3	1.99 (1.53, 2.45)	5.011 × 10^–17^	5.512 × 10^–16^	144.81	8.4	0.0146*	2.106 (1.865, 2.347)	3.88 × 10^–66^
SULT2A1	5alpha-pregnan-diol disulfate	10	1.16 (0.54, 1.78)	0.00025	0.00069	162.17	33.5	0.0001*	1.245 (0.753, 1.737)	0.007
SULT2A1	tauro-beta-tetrahydrocortisol sulfate	10	1.76 (1.12, 2.4)	9.3763 × 10^–8^	5.156 × 10^–7^	162.17	22.3	0.00793*	1.681 (1.109, 2.253)	0.0009
X-12410	Transferrin	3	−0.11 (−0.206, −0.014)	0.017	0.03	39.14	0.98	0.613	**-**	**-**
5alpha-androstan-3alpha,17alpha-diol monosulfate	RA51D	3	0.43 (0.299, 0.561)	1.66 × 10^–10^	1.49 × 10^–9^	115.45	18.22	0.0001*	0.463(−0.016,0.94)	4.06 × 10^–86^
tauro-beta-tetrahydrocortisol sulfate	RA51D	2	0.211 (0.107, 0.315)	8.4 × 10^–105^	0.0002	62.67	2.6	0.1	**-**	**-**
*ACADM*	3-decenoylcarnitine	2	3.12 (2.309, 3.931)	5.3 × 10^–14^	1.6 × 10^–13^	41.2	3.4	0.06	**-**	**-**
*CYP2D6*	solanidine	3	−0.361 (−0.671, −0.051)	0.022	0.03	95.03	4.4	0.11	**-**	**-**

FDR: False discovery rate

Q: Cohran’s Q statistic

Q_pval: Corresponding p-value assessing heterogeneity

^*^ Significant heterogeneity (P value < 0.05)

^±^ Radial MR was only performed if outlier variants were present

We further explored the results of the sensitivity analysis by performing a two-sample Mendelian randomization analyses using N2-acetyl-N6,N6-dimethyllysine as the exposure and diabetes as the outcome, deriving genetic instruments for diabetes from a recent large GWAS on type 2 diabetes [21]. These analyses indicated that genetically predicted non-missingness of N2-acetyl-N6,N6-dimethyllysine was associated with a reduced risk of type 2 diabetes (IVW:β = − 0.03, *p* = 0.04; Weighted median: β = − 0.03, *p* = 4.70 × 10^–5^). Evidence of heterogeneity across genetic instruments was observed (Cochran’s Q = 7.42, Q-*p* = 0.02); however, there was no indication of directional horizontal pleiotropy based on the MR-Egger intercept test (intercept = 0.10, SE = 0.04, *p* = 0.22). The estimates remained consistent using the MR-Radial method and no instrumental outliers were detected.

## Discussion

We conducted a genome-wide meta-analysis to identify genetic variants associated with the odds of missingness of metabolites and identified 55 lead SNP- ‘missing metabolite’ associations, of which 42 associations were novel (i.e., not found in previous GWAS of metabolite levels). Based on comprehensive in silico functional analyses, we identified associations between specific groups of metabolites and common metabolic pathways. First, we identified several SNP-metabolite associations involving metabolites that play a biological role in fatty acid beta oxidation pathways—generally containing an acetyl group—and that are also expressed or related to kidney function. Second, we found a group of metabolites and SNPs related to bile acid and steroid metabolism. Third, we identified SNPs associated with two xenobiotics primarily derived from plant sources.

Regarding the first group of SNP-metabolites associated with fatty acid beta oxidation, notable findings were related to the following metabolites:3-decenoylcarnitine, N-acetylkynurenine, N2-acetyl,N6,N6-dimethyllysine, and indoleacetylglutamine. This group of metabolites were overall associated with beta-oxidation, mitochondrial function, IEMs, and potentially related to kidney function.

3-decenoylcarnitine is a medium-chain acylcarnitine that is involved in the production of energy via beta-oxidation by transporting acyl-groups into mitochondria [23]. We identified five novel associations with 3-decenoylcarnitine’s odds of missingness in the intronic regions of *ACADM* and *ECI2,* in addition to an exonic loci in *PPID (*also known as *CypD * [24])*.* All five novel loci associated with 3-decenoylcarnitine were located in the vicinity of genes that were strongly linked to the regulation of fatty acid beta-oxidation [25–27]. In addition, disruptions of the metabolism of acylcarnitine and the process of beta-oxidation are well known causes for specific IEM. *ACADM* in particular is linked to medium chain acyl-CoA dehydrogenase (MCAD) deficiency [26, 28], supporting our hypothesis of genetic variations affecting the missingness of 3-decenoylcarnitine. Further evidence to support this hypothesis was also found in our study. First, the loci was only found in our missingness GWAS and not the quantitative GWAS or any previous GWAS studies for this metabolite. Second, our functional validation analyses using gene expression data showed that *ACADM* and *ECI2* expression were associated with their respective genetic variants, as well as with those of 3-decenoylcarnitine. This was further reinforced by the eQTL MR analysis that showed a strong potentially causal association between *ACADM* and the missingness of 3-decenoylcarnitine. Finally, the pQTL analysis using PhenoScanner showed that rs9410 (*PPID* gene) had been previously associated with PPID protein expression. The protein encoded by *PPID* (CypD) functions as a transport pore in the mitochondrial membrane [29], and plays an important role in the acetylation of proteins in the fatty acid oxidation and branched-chain amino acid metabolism, as reported in mice models [30]. Therefore, it is possible that mutations in this gene might affect the transport of 3-decenoylcarnitine in the mitochondria and lead to a reduction of its levels below the limit of detection, but further studies are required to substantiate this hypothesis. Additionally, the upstream metabolite carnitine had nearly no missingness in our data (none in NEO and only missing in 9 individuals in the Rhineland study). However, we observed that 3-decenoylcarnitine missingness occurred with higher carnitine levels, not when carnitine was low (Additional file 1: Fig. S3A and Additional file 1: Fig. S4A). This is an indication that these genetic variations could be affecting the expression or protein functions responsible for the metabolism or transport of carnitine to 3-decenoylcarnitine leading to an accumulation of carnitine and delayed production of 3-decenoylcarnitine.

N-acetylkynurenine is a metabolite belonging to the kynurenine pathway, which is crucial for the breakdown of tryptophan [31]. In our study we found that 3 loci were associated with its missingness. Among those, the locus in *ACY3* (rs948445), a gene encoding a deacetylation protein, was only associated with the missingness of N-acetylkynurenine and not the quantitative levels of the metabolite in our study (but was reported in a previous GWAS [10]). In addition, we identified a potentially causal association between this gene and the missingness of N-acetylkynurenine in the pQTL MR analysis, implying a role between the loci affecting the protein function, which in turn affects the deacetylation and the missingness of N-acetylkynurenine. Unlike it is paralog *ACY1, ACY3* was not reported to be associated with any IEM disorders [32]. We also found that the probability of N-acetylkynurenine missingness is linked to the SNP rs10188058 in the *STAMPB* gene. It is worth noting that both N-acetylkynurenine (as well as the kynurenine pathway) and *STAMPB* were previously reported to be associated with the IEM microcephaly-capillary malformation syndrome [31, 33]. The third locus (tagged by rs10201159) was located near *NAT8* and has been associated with the levels of acetyl forms on amino acids such as N-acetylglutamine [34]. These associations are expected as *NAT8* encodes an N-acetyltransferase [35]. In addition, *NAT8* is associated with kidney function and chronic kidney disease (CKD) progression. Specifically, genetic studies found an association between *NAT8*, higher levels of 15 acetylated amino acids (no association reported for N-acetylglutamine), and the progression of CKD [36]. Furthermore, *ACY3* [37, 38] and *STAMBP* [38, 39] have also been reported to be associated with CKD or eGFR. Further characterization of these loci in future studies may provide new insights into the role of N-acetylkynurenine and related pathways in CKD progression.

N2-acetyl,N6,N6-dimethyllysine is an amino acid and is the precursor of N6,N6,N6-trimethyl-L-lysine (also known as trimethyllysine (TML)), which in turn has been reported as a potential precursor for trimethylamine and trimethylamine N-oxide [40, 41]. Although little is known about N2-acetyl,N6,N6-dimethyllysine in the literature, its precursor—namely TML—have been studied extensively [40–42]. TML has been associated with cardiovascular diseases and reportedly predicted all-cause mortality and cardiovascular disease [41]. The loci associated with the missingness of N2-acetyl,N6,N6-dimethyllysine are near the regions of *PYROXD2* and *NAT8*. These two genes are related as *PYROXD2* has been reported to interact with *NAT8* in several studies [9, 43, 44]. *PYROXD2* is localized in the mitochondrial inner membrane and has an important role in regulating mitochondrial function [45]. In addition, *PYROXD2* is associated with the IEM disorder trimethylaminuria [46]. *PYROXD2* is normally associated with low levels of trimethylamine in the urine of healthy individuals [46]. Our PheWAS analysis indicated the lead SNP rs2147896 of N2-acetyl,N6,N6-dimethyllysine mapped to *PYROXD2* gene is associated with serum albumin measurement, which is a key indicator of kidney function. Although the commonly reported primary mutations causing this disorder are in the *FMO3* gene, growing evidence suggests that mutations in *PYROXD2* play a role as well [46, 47]. The eQTL analysis further supported that *PYROXD2* expression was associated with the SNPs (rs11189559 and rs2147896) and with the odds of N2-acetyl,N6,N6-dimethyllysine missingness. Our GWAS and eQTL findings could indicate the involvement of our novel SNPs associations in *PYROXD2* in relation to the missingness of N2-acetyl, N6,N6-dimethyllysine. In addition, our two-sample Mendelian randomization analysis using genetically predicted non-missingness of N2-acetyl-N6,N6-dimethyllysine (*ALMS1* and *PYROXD2*) as the exposure and T2D as the outcome indicated that the non-missingness of N2-acetyl-N6,N6-dimethyllysine is associated with a reduced risk of T2D (or, equivalently, that N2-acetyl-N6,N6-dimethyllysine missingness is associated with a higher risk of T2D). This could indicate that either (part) of the association of the respective genetic loci with the odds of missingness of this particular metabolite may be due to their association with diabetes, or vice versa. Interestingly, in a previous study, higher levels of N6, N6-dimethyllysine were found to be potentially causally associated with a lower risk of diabetic retinopathy [20], providing indirect support for the latter possibility. While this finding should be interpreted with caution, it suggests a potential link between this metabolite and diabetes-related metabolic pathways. Consequently, these associations may reflect poor metabolism of TML and trimethylamine, which in turn might be related to the development of trimethylaminuria. Furthermore, from our GWAS, eQTL, Mendelian randomization analyses, and the evidence from the literature, the missingness of N2-acetyl,N6,N6-dimethyllysine and the loci associated with its missingness could be associated with other comorbidities such as cardiovascular disease, CKD, and particularly T2D.

Finally, indoleacetylglutamine, a gut microbiome–derived metabolite [48] involved in tryptophan metabolism and has been reported in various studies regarding gut microbiome and chronic kidney disease [49, 50]. In our study, we identified two novel loci near the *ACSM1* and *ACSM2A* genes that were associated with the odds of missingness of this metabolite. Similar to *ACADM*, both *ACSM1* and *ACSM2A* are involved in the metabolism of acyl-CoA in medium-chain fatty acids. In addition, significant associations have been reported between genetic variants in these genes and eGFR and serum creatinine levels [51, 52], indicating a role in kidney function. This is further supported by their association with indoleacetylglutamine, which is part of the tryptophan metabolism pathway (shown in Fig. 3) that also involves N-acetylkynurenine and *NAT8,* which in turn are related to CKD.

The second group of metabolites we report was composed of steroids and bile acid metabolites with a shared association with two pleiotropic SNPs: rs4149056 and rs45446698. The rs4149056 (*SLCO1B1*) SNP was associated with two bile acids, two estrone metabolites, and an unknown metabolite X-12456—predicted to be analogous to steroid metabolites—which is in line with findings from previous genomic research literature [10]. First, *SLCO1B1* is associated with statin-induced myopathy via the interaction with bile acids and cholesterol [53]. Second, rs4149056 and *SLCO1B1* were reported to be associated with serum estrone levels [54, 55]. Similarly, rs45446698 (*CYP3A7*) has been reported in studies relating breast cancer to estrone and progesterone levels [56–58]. In addition, PheWAS analysis indicated previous associations between rs45446698 and birth weight, a trait with lifetime implications for metabolism [59]. Overall, these two SNPs participate in similar metabolic processes affecting steroid and bile acids. Although it remains unclear how these two SNPs are involved in reducing the metabolite levels to missingness levels, our pQTL suggests that rs45446698 is associated with post-translational modification of DNA repair protein RAD51 homolog 4 (RA51D) in a trans manner [60, 61]. This was further supported by our forward and reverse pQTL MR analysis. These modifications could indicate consequences of the SNP on the RAD51 functionality that could contribute to the missingness of estrone 3-sulfate, tauro-beta-muricholate, “5alpha-androstan-3alpha,17alpha-diol monosulfate”, and glyco-beta-muricholate.

The final group we have identified was comprised of alliin and solanidine, which are both xenobiotic metabolites derived from the consumption of garlic and potatoes, respectively. Alliin is generally known for its health benefits, such as improved glucose tolerance in mice and anti-inflammatory effects in rats and in vitro studies [62, 63]. Interestingly, the *NAT8* locus was found to be associated with the odds of missingness of alliin but not with the quantitative levels of this metabolite. In addition, a previous study reported a different SNP in *NAT8* that was associated with the acylated form of alliin—N-acetylalliin [10]. This novel finding indicates that the locus in *NAT8* engages in the metabolism and acylation of alliin in some capacity. It is possible that rs10201159 leads to a faster metabolism of alliin into N-acetylalliin. Consequently, the levels of alliin could fall below the detection limit and are therefore reported as missing in metabolomic analyses.

Solanidine is a steroidal alkaloid, a slightly toxic metabolite in low quantities derived from potatoes and other plants of the Solanaceae family [64, 65]. We identified three novel SNP associations from three genes strongly associated with solanidine. The rs2413667 SNP in the eQTL region of *CYP2D6* was particularly noteworthy. The coded protein from this gene is responsible for the metabolism of approximately 25% of drugs used in clinical settings and its association with solanidine has been studied in relation to metabolism efficiency [66, 67]. Solanidine has also been reported as a potential dietary marker to assess the efficiency of *CYP2D6* functionality [68]. This was further supported by a clinical trial reporting *CYP2D6* inhibition to be associated with up to a 4.56-fold increase in solanidine levels, indicating compromised xenobiotic metabolism, and additional studies used solanidine as a biomarker to identify “poor metabolizers” [66, 68, 69]. Based on our findings regarding the association of rs2413667 and the odds of missingness of solanidine—from the GWAS and eQTL MR analyses—in tandem with previous studies examining solanidine and *CYP2D6* metabolism, rs2413667 may be utilized as a new pharmacogenomic marker to identify poor metabolizers (or conversely rapid metabolizers) of drugs [70] and could be utilized in identifying and developing personalized nutritional interventions [71]. This may also be true for all the reported SNPs and metabolites we have identified, but would require further studies. The rs2413667 SNP is also in proximity to the eQTL region of *SMDT1,* and based on our mediation analysis, this eQTL has a suggestive significant mediation effect of 3.2%. The *SMDT1* encoded protein from this gene partakes in forming a calcium uniporter complex in the mitochondria. In line with the protein function, solanidine and solanine toxicity are characterized by the disruption of calcium transport in mitochondria [65]. Therefore, rs2413667 may affect solanidine metabolism through its influence on *SMDT1* expression.

Four pleiotropy patterns characterized the total 55 associations (Fig. 3). First, the odds of missingness of 13 metabolites were associated with multiple loci in different genes, as was the case, e.g., for 3-decenoylcarnitine. Second, five loci illustrated pleiotropy and were associated with the odds of missingness of multiple metabolites, usually belonging to similar metabolic pathways. A noteworthy case of this was rs4149056, which was associated with the odds of missingness of five metabolite measures—three of which were steroid metabolites. Third, we observed three instances where multiple SNPs within the same gene were associated with the odds of missingness of corresponding metabolites. For example, three SNPs in *PYROXD2* were associated with the odds of missingness of N2-acetyl,N6,N6-dimethyllysine. Fourth, the remaining associations were exclusively single SNP-metabolite associations. Taken together, our findings indicate considerable genetic pleiotropy regarding the odds of missing metabolite measures, which, however, converge on common metabolic pathways.

An important consideration for this study is the interpretation of results originating from a non-traditional phenotype—the missingness of metabolites. Missingness of a metabolite measurement can be caused by either a technical issue, i.e., failure of metabolite identification in the spectral data due to a deconvolution issue, the metabolite concentration being below the limit of detection, or real missingness, i.e., the metabolite concentration is null. It can be expected that failure of metabolite detection in the spectral data due to a deconvolution issue is, to an extent, random and unlikely to be caused by genetics. However, it is extremely difficult to distinguish between a metabolite measure being below the limit of detection and a metabolite truly being absent, such as the case of dopamine 4-sulphate. The SNP rs67110785 is found to be associated with the missingness of dopamine 4-sulphate measures and is located in close proximity to the tyrosine hydroxylase (TH) encoding gene—a rate-limiting enzyme in the synthesis of dopamine [72]. The same SNP is also an eQTL for *TH* in the Genotype-Tissue Expression (GTEx) database. At face value, TH deficiency would lead to missingness of dopamine 4-sulphate; however, TH deficiency also leads to severe neurological problems that were not reported by any of the participants. Dopamine 4-sulphate is produced from dopamine by the enzyme sulfotransferase family 1 A member 3 (SULT1A3), which also produces dopamine 3-sulphate [73]. When plotting the levels of dopamine 4-sulphate against dopamine 3-sulphate in the NEO study, it was clear that the individuals with missing values of dopamine 4-sulphate had low levels of dopamine 3-sulphate and could thus not be TH or SULT1A3 deficient (Additional file 1: Fig. S3B and Additional file 1: Fig. S4B). The missingness of dopamine 4-sulphate is therefore likely due to the measures being below the limit of detection, probably caused by lower levels of dopamine, rather than its absence.

Although the exact mechanism through which these SNPs would induce the missingness of metabolites remains to be fully elucidated, findings from previous studies related to IEM and poor metabolism, as well as our eQTL and pQTL analyses, support the hypothesis that genetic factors influence the probability of metabolites' absence. Future studies are needed for a deeper investigation of the underlying biological pathway of the missingness of the reported metabolites. A limitation of our study is the relatively small sample sizes used in the GWAS. However, despite this limitation we were able to identify strong associations in both studies. Furthermore, by using two studies and a meta-analysis approach, we replicated the associations between the genetic variants and missingness. We expect that with a larger sample size even more associations will be identified. A second potential limitation was using different blood sample collection methods, with serum used in NEO and plasma in the Rhineland study. The differing blood sampling methodology could have influenced the levels and detection of some metabolites and may explain some of the disparity in the total measured metabolites between the two studies. However, we did find a very strong correlation between the loci-metabolites effect estimates from the NEO and Rhineland study. Additionally, high correlations were previously reported for metabolite measurements from serum and plasma samples collected from the same individuals [74]. Therfore, the choice of blood sampling type may have a limited impact on the overall metabolite profiles and our findings. A third limitation was the nature of the untargeted platforms. These platforms can be prone to missing data due to systematic errors [12, 13]. We accounted for this limitation by excluding metabolites outside the missingness limits (< 10% or > 90% missingness) to avoid the inclusion of metabolites that were simply missing due to systematic errors as much as possible. Future targeted metabolomics studies that measure the absolute concentrations of our reported metabolites can aid in replicating our findings. Finally, our study included populations from European ancestry only and, therefore, further studies are required to investigate metabolite missingness in different populations and ethnicities.

## Conclusions

In summary, we identified 55 associations between genetic variants and the odds of missingness of numerous metabolites, 42 of which were completely novel associations. These associations involved 24 SNP-metabolite pairs related to fatty acid beta oxidation and kidney function. In addition, two pleiotropic SNPs were notable for their associations with metabolites partaking in steroid and bile acid metabolism, as well as metabolism of dietary and xenobiotic metabolites. Our results provide novel insights into the role of genetics in determining the absence of certain metabolites, with potential implications for the identification of both “poor metabolizers” and IEM. Indeed, the novel genetic variants reported here could have potential value in future etiological and prediction studies, especially in the fields of metabolomics, nutritional epidemiology, pharmacogenomics, and IEM disorders.

## Methods

### Study populations

We included 594 and 4,165 individuals of European ancestry from the Netherlands Epidemiology of Obesity (NEO) study and the Rhineland Study, respectively, who had complete genetic and metabolomics data. NEO is a population-based, prospective cohort study initiated in 2008. All participants recruited in this study gave written informed consent, and the Medical Ethical Committee of the Leiden University Medical Center (LUMC) approved the study design. A detailed description of the study design and data collection can be found elsewhere [75]. Briefly, men and women aged between 45 and 65 years with a self-reported body mass index (BMI) of 27 kg/m^2^ or higher living in the greater area of Leiden (in the west of the Netherlands) were eligible to participate in NEO. Participants were invited for a baseline visit at the NEO center in the LUMC after an overnight fast. At the baseline visit, fasting blood samples were drawn. The Rhineland Study is an ongoing prospective population-based cohort study in Bonn, Germany. People aged 30 years or above who lived in two geographically defined areas in Bonn were invited to participate, with the only exclusion criterion being insufficient command of the German language to provide informed consent. Each participant undergoes a comprehensive 7-h examination protocol, which includes detailed questionnaires, brain imaging, neuropsychological examinations, and blood sample analyses. Prevalent diabetes was defined based on the current use of antidiabetic medication (ATC code A10), glycated hemoglobin (HbA1c ≥ 6.5%), fasting plasma glucose (≥ 126 mg/dL), or self-reported physician-diagnosed diabetes obtained during the medical interview. HbA1c < 6.5% and fasting plasma glucose < 126 mg/dL were considered indicative of no diabetes. Data collection in the Rhineland Study was approved by relevant ethics committees and/or institutions and was conducted according to the Declaration of Helsinki. All participants provided written informed consent.

### Metabolomics measurements and missingness inclusion criteria

Metabolites were measured on the Metabolon HD4 platform in the fasting state serum samples (*N* = 594) from NEO and fasting state plasma samples (*N* = 4,165) from the Rhineland Study. Only very few samples were collected in a non-fasting state (*N* = 51). Details on the metabolomics pipeline have been described elsewhere [11]. In brief, the Metabolon HD4 platform employs an untargeted measurement approach that utilizes reverse phase ultra-performance liquid chromatography (UPLC) tandem mass spectrometry (MS/MS) combined with a positive ion mode electrospray ionization, RP-UPLC-MS/MS combined with a negative ion mode electrospray, and hydrophilic interaction liquid chromatography (HILIC)-UPLC-MS/MS combined with a negative ion mode electrospray ionization. In total, 1,365 metabolites were measured in NEO, and 1,077 were measured in the Rhineland Study. Of these, 847 and 467 were endogenous in NEO and the Rhineland Study, respectively. Based on the pathway annotations by Metabolon, these endogenous metabolites spanned 10 pathway groups: amino acids, cofactors and vitamins, lipids, energy, nucleotides, peptides, carbohydrates, and partially characterized molecules. In addition, it included measurements of 222 and 321 xenobiotic metabolites as well as 296 and 289 unannotated metabolites from NEO and the Rhineland Study, respectively.

Most of the missingness for endogenous metabolites measured by the metabolon platform occurs in less than 10% of the measured population. These cases are commonly due to systematic or random errors in measurements. On the other hand, the majority of xenobiotic metabolites are missing in at least 90% of the population as these metabolites depend on specific external exposures (i.e., medication use, nicotine exposure, etc.). Therefore, we included metabolites with a moderate number of missing values by excluding metabolites that had a missingness percentage that was either below 10% or above 90% within each study. The rationale for this approach was to exclude metabolites with a high probability of having missing values due to systematic and random errors (< 10%), that are truly missing (> 90%), or other unknown causes. Accordingly, we selected 341 in the NEO study and 425 metabolites in the Rhineland Study.

### Genotyping and imputation

In NEO, DNA was extracted from 6,671 venous blood samples obtained from the antecubital vein. Genotyping was performed in the Centre National de Génotypage (Evry Cedex, France), using Illumina HumanCoreExome-24 BeadChip (Illumina Inc., San Diego, California, United States of America). The detailed quality control process has been described previously [76]. Genotypes were imputed to the Haplotype Reference Consortium (HRC) release 1.1 [77]. In the Rhineland Study, 4,165 DNA samples isolated from buffy coats extracted from blood samples were genotyped using the Illumina Omni-2.5 exome array and processed with GenomeStudio (version 2.0.5). Quality control was performed using PLINK (version 1.9). SNPs were excluded based on poor genotyping rate (< 99%) or Hardy–Weinberg Equilibrium (*p* < 1 × 10^–6^). Additionally, participants with poor quality DNA samples were excluded, on account of a poor call rate (< 95%) (*N* = 41), abnormal heterozygosity (*N* = 69), cryptic relatedness (*N* = 261), or a sex mismatch (*N* = 28). To account for variation in the population structure, which may otherwise cause systematic differences in allele frequencies, we used EIGENSTRAT (version 16,000), a principal component-based analysis to assess population structure [78]. In addition, prior to the GWAS, individuals of non-European ancestry were identified based on the clustering of their genetic principal components relative to the main European ancestry cluster and were excluded from further analyses. To ensure a genetically homogeneous study population of European ancestry, 164 individuals who fell outside the PCA-defined European ancestry cluster were removed. Finally, imputation was performed with IMPUTE (version 2) [79], using the 1000 Genomes version 3 phase 5 as the reference panel [80]. We sought a high imputation quality by only including SNPs with an R-squared (R2) info metric > 0.3, which indicates reliable imputation [81]. After quality control, all autosomal SNPs passing these criteria—representing over 95% of common variants (MAF > 0.01)—were retained for the subsequent analyses.

### Genome-wide association analyses

In NEO, we performed the GWAS of missing metabolites using the SNPTEST v2 software, employing logistic regression analysis under an additive model. In the Rhineland Study, the GWAS was performed using the REGENIE software (v2.2) [82], fitting a firth logistic regression model to the data. REGENIE computation is composed of two steps. Step 1 uses a subset of genetic markers to fit a whole genome regression model that captures the phenotype variance attributable to genetic effects. In step 2, a larger set of imputed SNPs are used in order to test for their association with the different phenotypes, conditional upon the prediction from step 1 and using a leave-one-chromosome-out scheme. Genotyped SNPs were pruned using a linkage disequilibrium (LD) r^2^-threshold of 0.9 with a window size of 1,000 and a step size of 100 markers.

Overall, we included 21,243,072 and 49,953,404 imputed SNPs in the GWAS analysis in NEO and the Rhineland Study, respectively. These analyses were restricted to SNPs with an imputation quality > 0.3 and minor allele frequency (MAF) > 0.01. The missing metabolites were adjusted for age, sex, fasting status, and five genetic principal components in the NEO study, and age, sex, fasting status, and the first ten genetic principal components in the Rhineland Study. Genetic principal components were included to control for residual confounding due to population structure, as they capture ancestry-related variation through linear combinations of genetic markers and are therefore standard in GWAS. Because analyses were restricted to individuals of European ancestry and additionally adjusted for genetic principal components, residual confounding due to population stratification is expected to be minimal. Genome-wide significance level was set at *p* < 5 × 10^–8^ [83]. However, because of the large number of outcome variables (i.e., missing metabolites), we corrected for multiple testing using the method of Li & Ji [84], which estimates the effective number of independent tests. Accordingly, we estimated the effective number of independent tests to be 315 and 313 in the NEO and the Rhineland Study, respectively, resulting in a metabolome-wide significance level of *p* < 1.58 × 10^–10^ (≈ 5 × 10^–8^/315) and *p* < 1.59 × 10^–10^ (≈ 5 × 10^–8^/313), respectively. As a sensitivity analysis, we additionally adjusted for prevalent diabetes (*N* = 205) in the Rhineland Study to assess the robustness of associations after accounting for diabetes status.

### Quantitative GWAS sensitivity analysis

To assess the uniqueness of the associations identified through GWAS on missing metabolites, we additionally performed a GWAS for the quantitative measures of the metabolites in the Rhineland study that were significant in the primary binary GWAS (26 metabolites). We used the same *p* < 1.59 × 10^–10^ cutoff as the binary GWAS as well. Missing values in these metabolites were left as is. We used REGENIE software (v2.2) [82], and used linear regression for association testing. Prior to association testing, metabolite levels were adjusted for age, sex, fasting status, batch effects, and the first ten genetic principal components using linear regression. The residuals from these models were then transformed using a rank-based inverse normal transformation to approximate a normal distribution. These transformed residuals were used as input phenotypes in the GWAS performed with REGENIE (v2.2).

### Assessment of potential study-specific effects

To assess potential study-specific effects, we performed SNP × study interaction analyses using the GWAS summary statistics for each study for each metabolite (224 metabolites). Heterogeneity in SNP effect estimates between the two studies was tested using EasyStrata [85]. We considered an interaction effect to be metabolome-wide significant if it attained a 1.59 × 10^–10^.

### Meta-analysis of GWAS

For the GWAS meta-analysis, we selected and used 7,310,783 SNPs that had MAF > 0.01 in the Rhineland Study, as it had the larger sample size between the two studies. We then harmonized the SNPs with the overlapping SNPs in the NEO study that had a MAF > 0.01 (6,610,552/7,310,783 SNPs). Meta-analysis was performed employing an inverse variance-weighted fixed-effects model using METAL [86]. Identification of allele flips and applying genomic control was performed using METAL as well for each cohort prior to performing the meta-analysis. The metabolome-wide significance level for the meta-analysis was set at p < 1.59 × 10^–10^, which was the more stringent cut-off used in the Rhineland Study. To verify if our findings for missing metabolites were indeed novel, we used curated metabolome GWAS databases such as mGWAS-Explorer [87] and PhenoScanner [88]. We further manually verified whether our lead SNPs were previously identified in metabolomic-based GWAS studies [10] as metabolome quantitative trait locus (mQTLs).

### Definition of genomic risk loci

To identify genomic regions associated with missing metabolites, a single dataset was created by identifying the minimum p-value for each SNP across all meta-GWAS summary statistics of missing metabolites. This dataset was LD-clumped (r2 < 0.6) using the Functional Mapping and Annotation (FUMA) platform [89] with the 1000 Genomes Phase 3 European reference panel to account for the LD structure. We further represented those clumped SNPs by lead SNPs, which are a subset of the independent significant SNPs that are in approximate LD with each other at r^2^ > 0.1. Finally, we identified associated genomic risk loci by merging any physically overlapping lead SNPs (LD blocks < 250 kb apart).

### Identifying candidate genes

We aimed to identify candidate genes tagged by the lead SNPs that may influence the probability of missingness of certain metabolites. To achieve this, we first used the “prioritization of candidate causal genes at molecular QTLs” (ProGeM) framework [90]. This framework implements a two-step approach (bottom-up and top-down) to identify putative causal genes. In the bottom-up approach, the three closest protein-coding genes within 500 kb of the lead SNP are selected, while the top-down approach uses curated gene function databases (e.g., Gene Ontology (GO), Kyoto Encyclopedia of Genes and Genomes (KEGG), Mouse Genome Informatics (MGI) and Orphanet) to identify biologically relevant genes that are present within 500 kb of the lead SNPs. We used Variant Effect Predictor (VEP) [91] to search for the closest protein coding genes with the lead SNP and also calculated the impact factor score of the lead SNPs based on their function as either missense, start loss, or stop gain. ProGEM also assesses whether the lead SNPs are eQTL using the GTEX v8 database [92]. The genes that were identified through the top-down and bottom-up approach were prioritized as candidate genes.

### PheWAS

We performed a phenome-wide association study (PheWAS) using the disgenet2r package to check which lead SNPs had previously been reported to be associated with any other clinical or disease outcomes, as contained in the disease-gene association database (DisGeNET). The package ranks the associations using Variant Disease Association (VDA) scores ranging from 0 to 1, where a higher score represents stronger evidence of a SNP association with a disease outcome. Only lead SNPs with a score > 0.7 were reported.

### RNA sequencing data in the Rhineland study

Total RNA was isolated from 3,384 whole blood samples in the Rhineland Study. Those samples were stored and stabilized in PAXgene Blood RNA tubes (PreAnalytix/Qiagen) using PAXgene Blood miRNA Kit in accordance with the manufacturer’s instructions and following the semi-automatic purification protocol (PreAnalytix/Qiagen). RNA integrity and quantity were assessed using the Tapestation 4200 system (Agilent). After using 750 ng of total RNA to generate next generation-sequencing libraries for total RNA sequencing (TruSeq stranded total RNA kit, Illumina), a Ribo-Zero Globin reduction was performed. Libraries were quantified using Qubit HS dsDNA assay (Invitrogen) and clustered at 250 pM concentrations on a NovaSeq6000 instrument using NovaSeq S2 v1 chemistry (Illumina) in XP mode for the first batch of 3,000 samples and NovaSeq S4 v1.5 chemistry for the last batch of 384 samples and sequenced paired-end 2 × 50 cycles. Sequencing data was demultiplexed and converted into fastq format using bcl2fastq2 v2.20. We performed the quality control check on raw sequencing reads using FastQC v0.11.9, and we filtered low-quality score reads using Trimmomatic v.0.39. Next, we used STAR v2.7.1 aligner to align the sequencing reads to the human reference genome GRCh38.p13 and to generate the gene count matrix through “STAR –quantMode GeneCounts” using the human gene annotation version GRCh38.101. Genes with overall mean expression greater than 15 reads and expressed in at least 5% of the participants were selected for further analysis. Finally, we used the “varianceStabilizingTransformation” function from DESeq2 v1.30.1 to normalize and transform the raw counts.

### Gene expression quantitative trait loci

To functionally validate the GWAS results using gene expression data, we used a three-step approach analysis leveraging data from the first 3,384 consecutive participants of the Rhineland Study, on whom genotype, gene expression, and metabolite data were available and passed quality control (*N* = 2,575). First, we assessed the associations between the lead SNPs and the corresponding genes, selected through bottom-up and top-down approaches. Each lead SNP was analyzed separately in relation to its corresponding candidate gene as the outcome. We adjusted gene expression levels for age, sex, the first 10 principal components, red and white blood cell counts, the relative fractions of basophils, eosinophils, lymphocytes, monocytes, and neutrophils, and batch effect, and we extracted the residuals. Next, linear regression analysis was performed to assess the associations between the lead SNPs (independent variable) and the residuals of candidate genes (dependent variable). Second, we evaluated the relations between the residuals of gene expression data, obtained after adjustment for identical covariates as before (independent variable), and the significant metabolites with missing values, adjusting for the metabolomics’ batch effect using logistic regression analysis. Third, we employed a mediation analysis using the R package lavaan v.06–11 to investigate which candidate genes mediated the associations between lead SNPs and missing metabolite with 1000 bootstrapping iterations.

### Protein quantitative trait analysis

We performed a protein quantitative trait analysis by using the Phenoscanner [88]. Briefly, Phenoscanner holds publicly available protein quantitative trait loci (pQTLs) results from large-scale genome-wide association studies. We verified whether our lead SNPs associated with missing metabolites were previously identified as pQTLs. Accordingly, we filtered results for protein wide association studies with significant SNP-protein associations (*p* < 5 × 10^–8^) with our lead SNP or a proxy SNP (r2 > 0.9) with our lead SNPs.

### Network representation of gene-SNP-metabolite associations

We used the associations identified through our meta-analysis, ProGEM mapping, and PheWAS to construct a comprehensive interactive network. Each SNP, metabolite, gene, metabolite sub-pathway (as annotated in the metabolon dataset), disease, or phenotype associations from DisGeNET, and pQTL associations from Phenoscanner were presented as “nodes” with distinct colours. The width of the “edges” connecting the SNP-metabolites was determined according to the -log10 of the p-value of the effect estimate. Additionally, the colour of these edges was chosen to reflect the novelty of the association. Visualization and layout of the networks were created using Cytoscape version 3.10.2 and Gephi v0.10 and then exported as an interactive HTML5 using the sigmaExporter plugin [93].

### Mendelian randomization of eQTLs and pQTLs

We evaluated the potential causal effects for the genes we identified on the missingness of the metabolites reported from the GWAS analyses using Mendelian randomization analysis (MR). For each gene, we used SNPs for their eQTL and pQTL data. We first used whole blood eQTLs from GTEX v8 as genetic instruments, treating gene expression as the exposure and missing metabolite phenotypes as the outcomes. Instruments were selected using LD clumping with a 10 Mb window, an LD threshold of r^2^ < 0.01, and a significance threshold of *P* < 1 × 10⁻^4^. We used the Two Sample Mendelian Randomization (MR) R package (v 0.6.4) [94]. Second, we conducted two-sample MR for the pQTL data per gene, which were used as instruments to assess the causal effect of protein levels on metabolite missingness. We applied the same clumping parameters with a stricter significant threshold of *P* < 5 × 10^–8^. Additionally, as a sensitivity analysis, we performed reverse MR analyses to evaluate whether the missingness of specific metabolites could causally influence protein expression. For all MR analyses, we performed MR Egger, simple mode, weighted median, weighted mode, Wald ratio, and inverse variance weighted (IVW) analyses—focusing on the IVW for interpretation. We also assessed the heterogeneity using Cochran’s Q and horizontal pleiotropy using the MR-egger intercept. Bias due to variant outliers was accounted for using Radial MR. Briefly, this method applies a simulation-based approach to detect outlying variants and remove them to re-estimate the associations. This analysis was performed using the Radial MR R package (v 1.1) [95].

## Supplementary Information

Additional file 1: Figs. S1-S4. Supplementary figures showing quantitative GWAS sensitivity analyses, correlation of SNP effect estimates between NEO and Rhineland study cohorts, and visualization of missingness of 3-decenoylcarnitine and dopamine 4-sulphate in relation to carnitine and dopamine 3-sulphate in NEO and Rhineland study cohorts

Additional file 2: Table S1. Genomic risk loci of metabolome wide significant SNPs in the NEO study

Additional file 3: Table S2. Genomic risk loci of metabolome-wide significant SNPs in the Rhineland Study

Additional file 4: Table S3. SNP*SNP interaction analyses using EasyStrata

Additional file 5: Table S4. Frequency of missing metabolites in the NEO study and Rhineland Study

Additional file 6: Table S5. Overall SNPs in linkage disequilibrium with lead SNPs identified in meta-analysis

Additional file 7: Table S6. Comparison of genomic risk loci identified in the GWAS-meta analysis with cohort specific GWAS results from the NEO and Rhineland Study cohorts

Additional file 8: Table S7. Overview of the lead SNPs in GWAS-meta analysis

Additional file 9: Table S8. Association of lead SNPs with inter and intra class metabolites

Additional file 10: Table S9. Lead SNPs look ups with protein quantitative trait lociusing PhenoScanner V2

Additional file 11: Table S10. Lead SNPs look ups with metabolone quantitative trait lociusing PhenoScanner V2, mGWAS and Praveen et.al database

Additional file 12: Table S11. ProGeM analysis of identifying positional genes using bottom-up approach for the lead SNPs

Additional file 13: Table S12. ProGeM analysis of identifying candidate genes using top-down approach for the lead SNPs

Additional file 14: Table S13. Functional validation analyses using gene expression data

Additional file 15: Table S14. DisGeNet phenome-wide association studyanalyses of lead SNPs

Additional file 16: Table S15. Two sample Mendelian Randomizationof gene expression and missing metabolites

Additional file 17: Table S16. Twosample MR of pQTLs and missing metabolites

## Data Availability

To protect participant privacy and comply with legal regulations, the NEO study data is not publicly accessible. Qualified researchers can request access by contacting the NEO Executive Board at https://www.lumc.nl/org/neo-studie/contact/ and https://www.lumc.nl/en/afdelingen/Clinical-Epidemiology/obesity--related-diseases/. Similarly, the Rhineland Study https://www.rheinland-studie.de/en/ data used in this manuscript is restricted to public access due to data protection laws. Researchers seeking access to these datasets can submit requests to RS-DUAC@dzne.de, providing evidence of their qualifications and adherence to the respective study's data use policies. All authors had full access to the data from their respective studies and are responsible for the accuracy and integrity of the data and analysis. The summary statistics of the meta-analysis GWAS are publicly available via the GWAS catalog under accession number GCP001677.
