# Supplementary material for: Genome-wide association studies of missing metabolite measures from two population-based studies
Source: Genome Biol. 2026 Jul 10;27:220. doi: 10.1186/s13059-026-04132-9 (PMC13352909; doi:10.1186/s13059-026-04132-9)

Fig. S1. Mirror Manhattan plot of missing vs quantitative metabolite GWAS

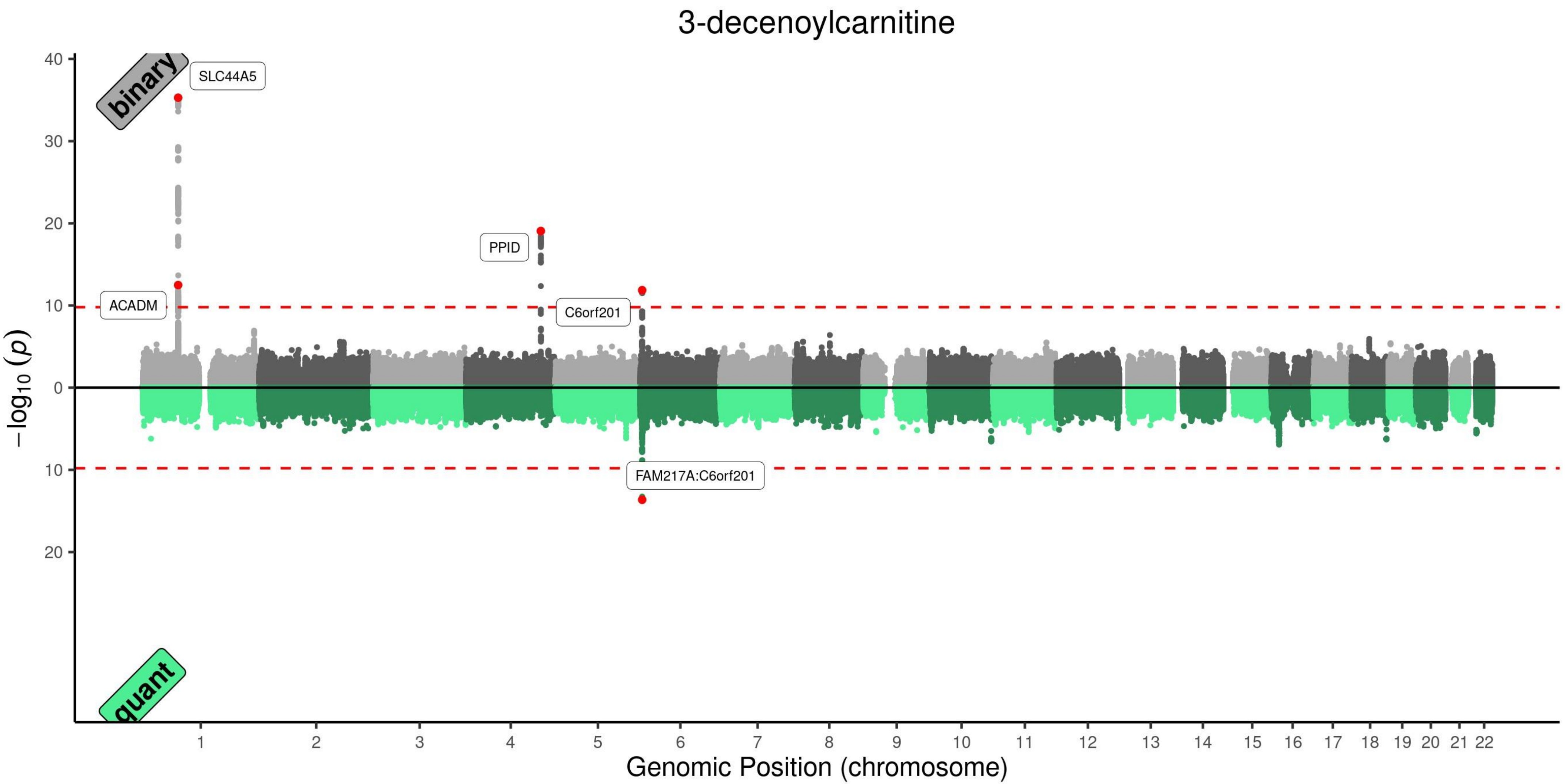

5alpha-androstan-3alpha,17alpha-diol monosulfate

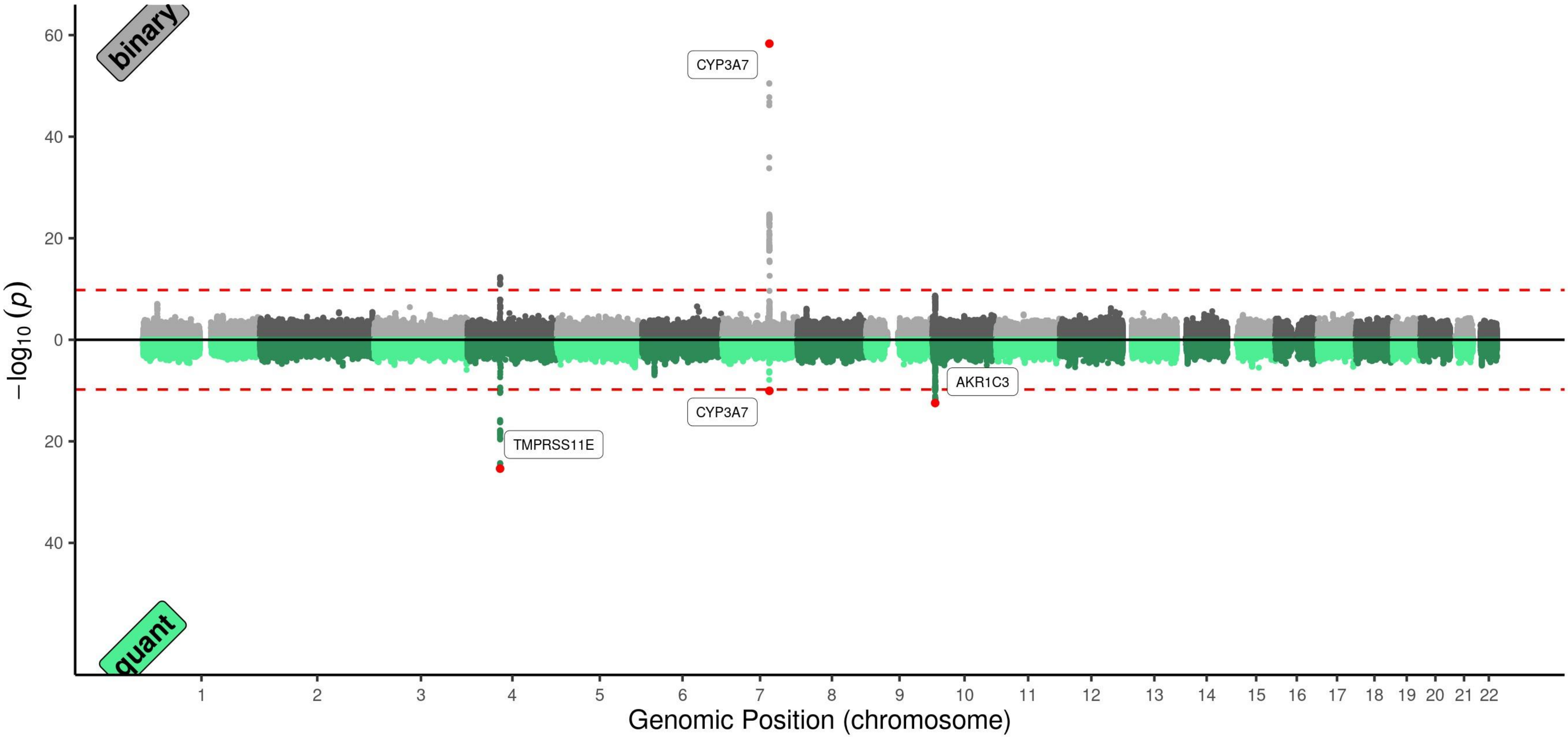

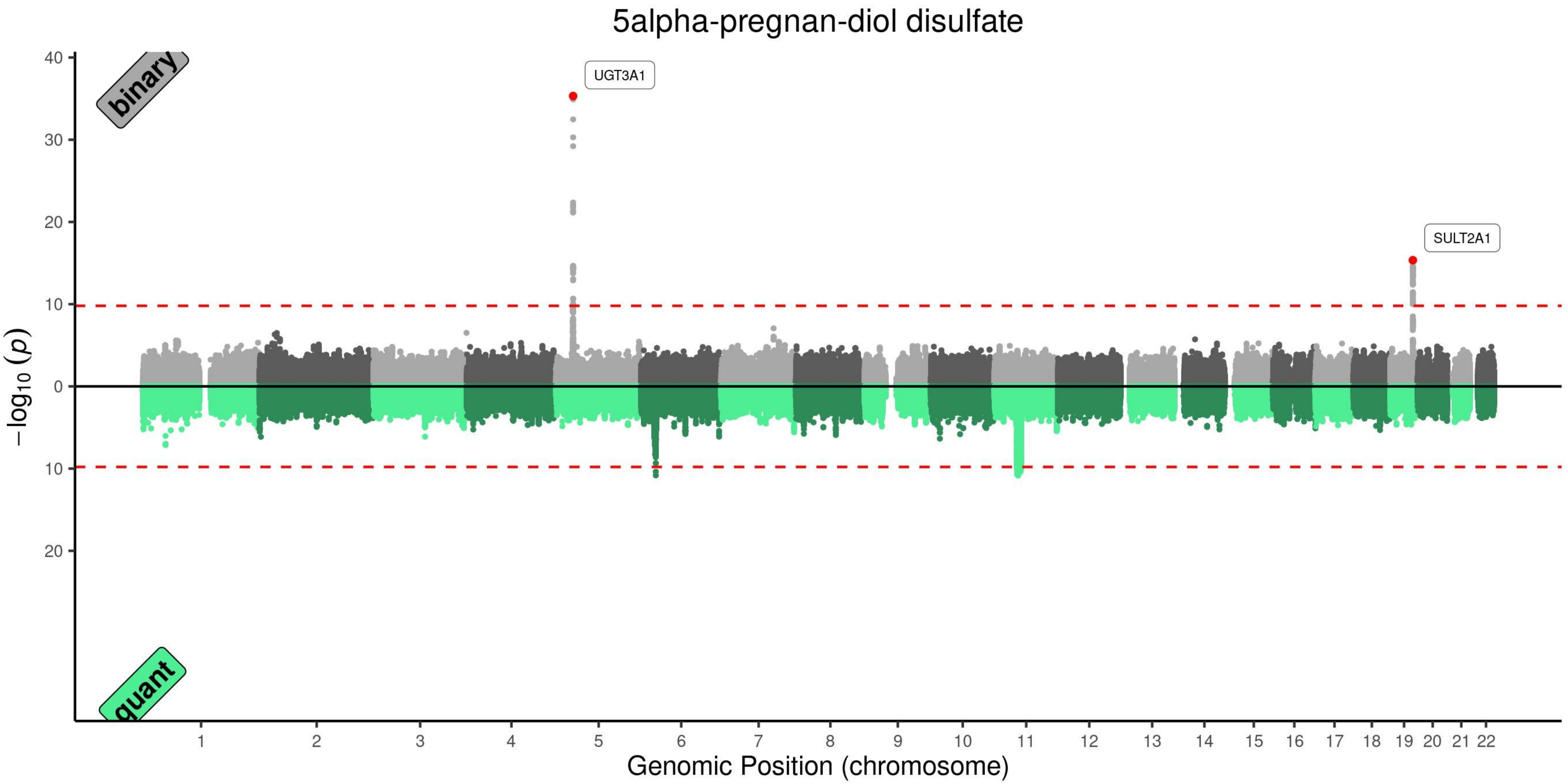
5alpha-pregnan-diol disulfate
binary
quant
UGT3A1
SULT2A1
$-\log_{10}(p)$
40
30
20
10
0
10
20
1
2
3
4
5
6
7
8
9
10
11
12
13
14
15
16
17
18
19
20
21
22
Genomic Position (chromosome)

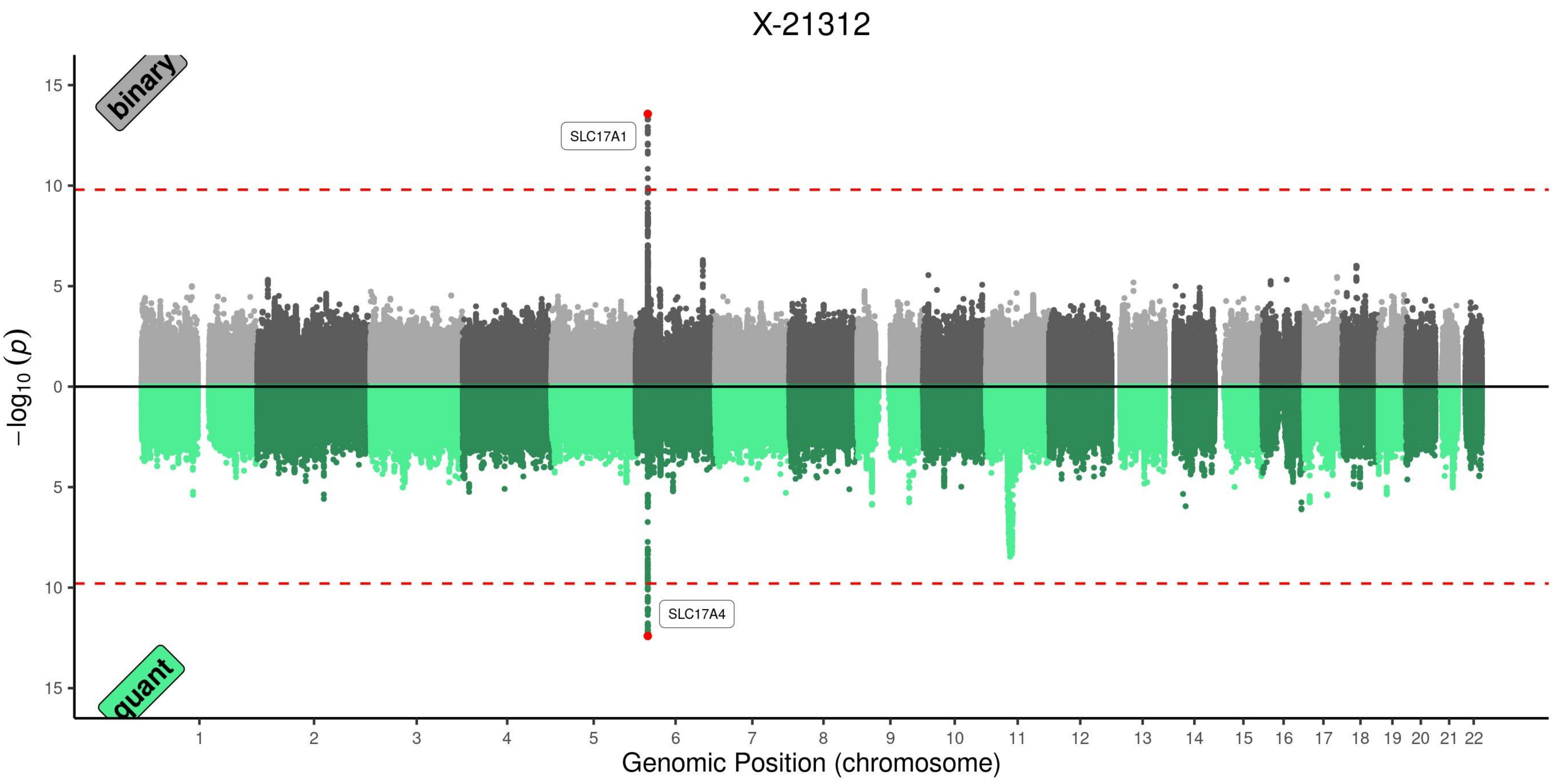
X-21312
binary
quant
SLC17A1
SLC17A4
15
10
5
0
5
10
15
$-\log_{10}(p)$
1
2
3
4
5
6
7
8
9
10
11
12
13
14
15
16
17
18
19
20
21
22
Genomic Position (chromosome)

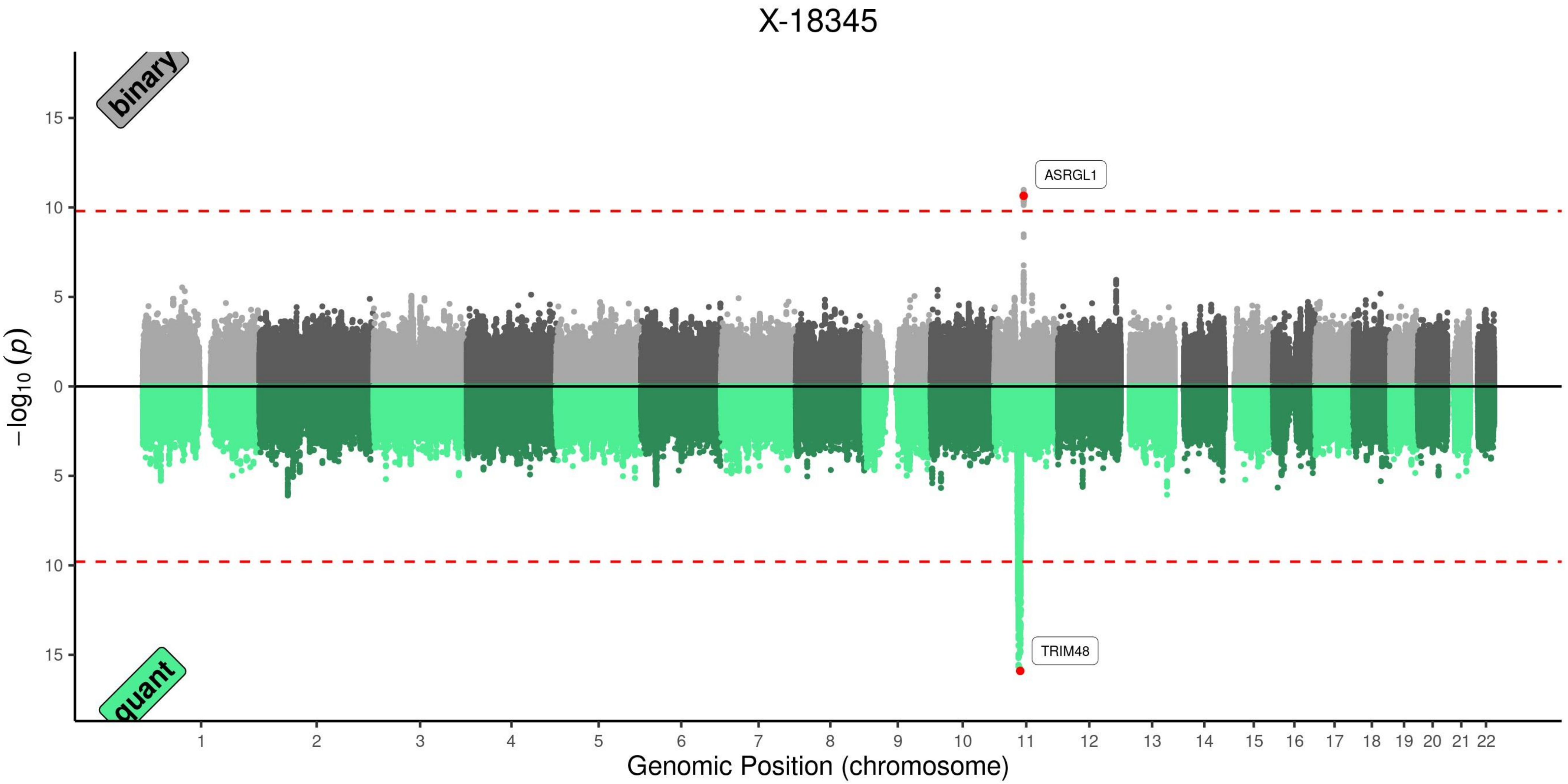

X-18345
binary
quant
ASRGL1
TRIM48
15
10
5
0
5
10
15
−log10(p)
1
2
3
4
5
6
7
8
9
10
11
12
13
14
15
16
17
18
19
20
21
22
Genomic Position (chromosome)

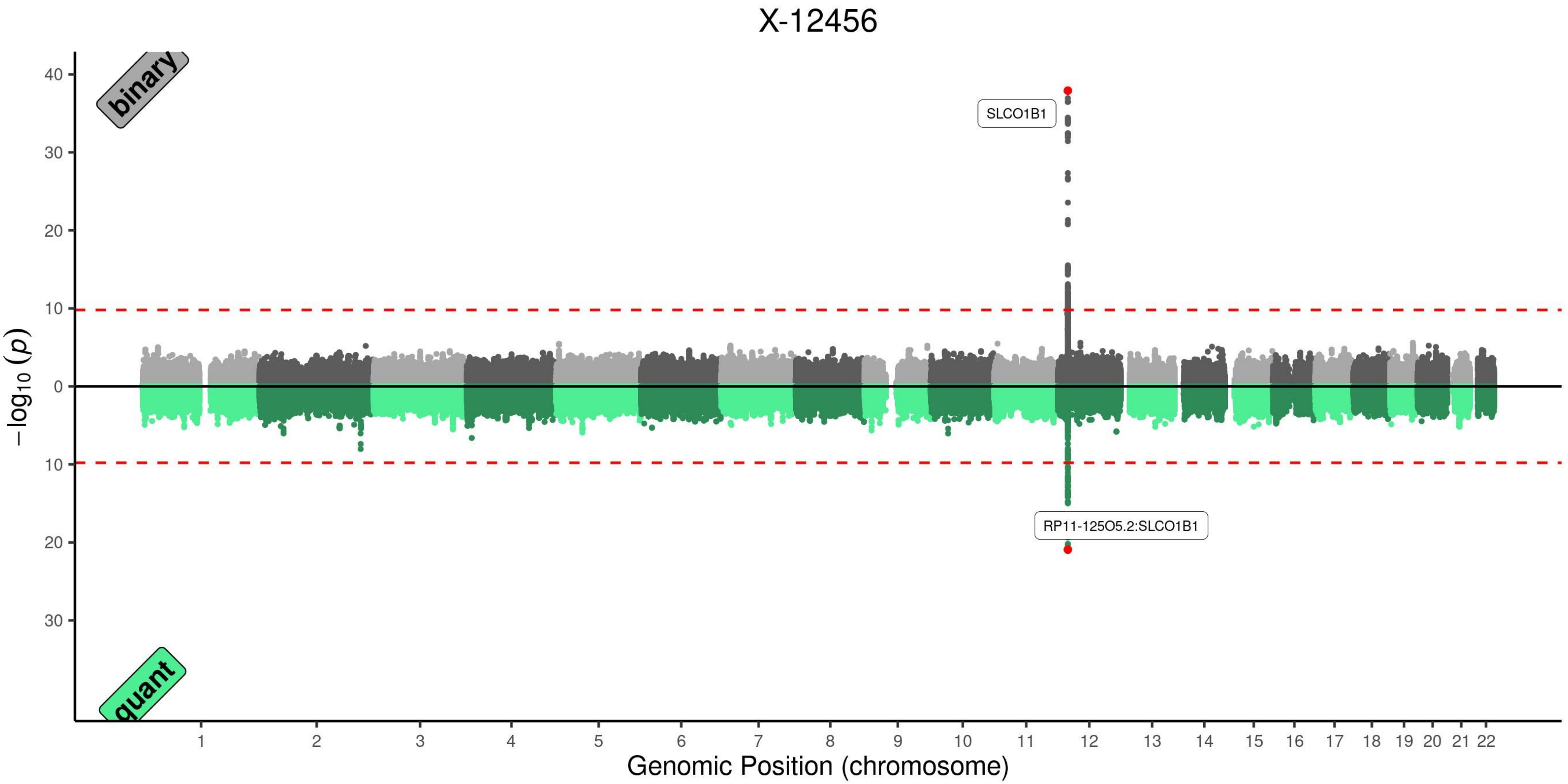

X-12456
binary
quant
SLCO1B1
RP11-125O5.2:SLCO1B1
−log10 (p)
40
30
20
10
0
10
20
30
1
2
3
4
5
6
7
8
9
10
11
12
13
14
15
16
17
18
19
20
21
22
Genomic Position (chromosome)

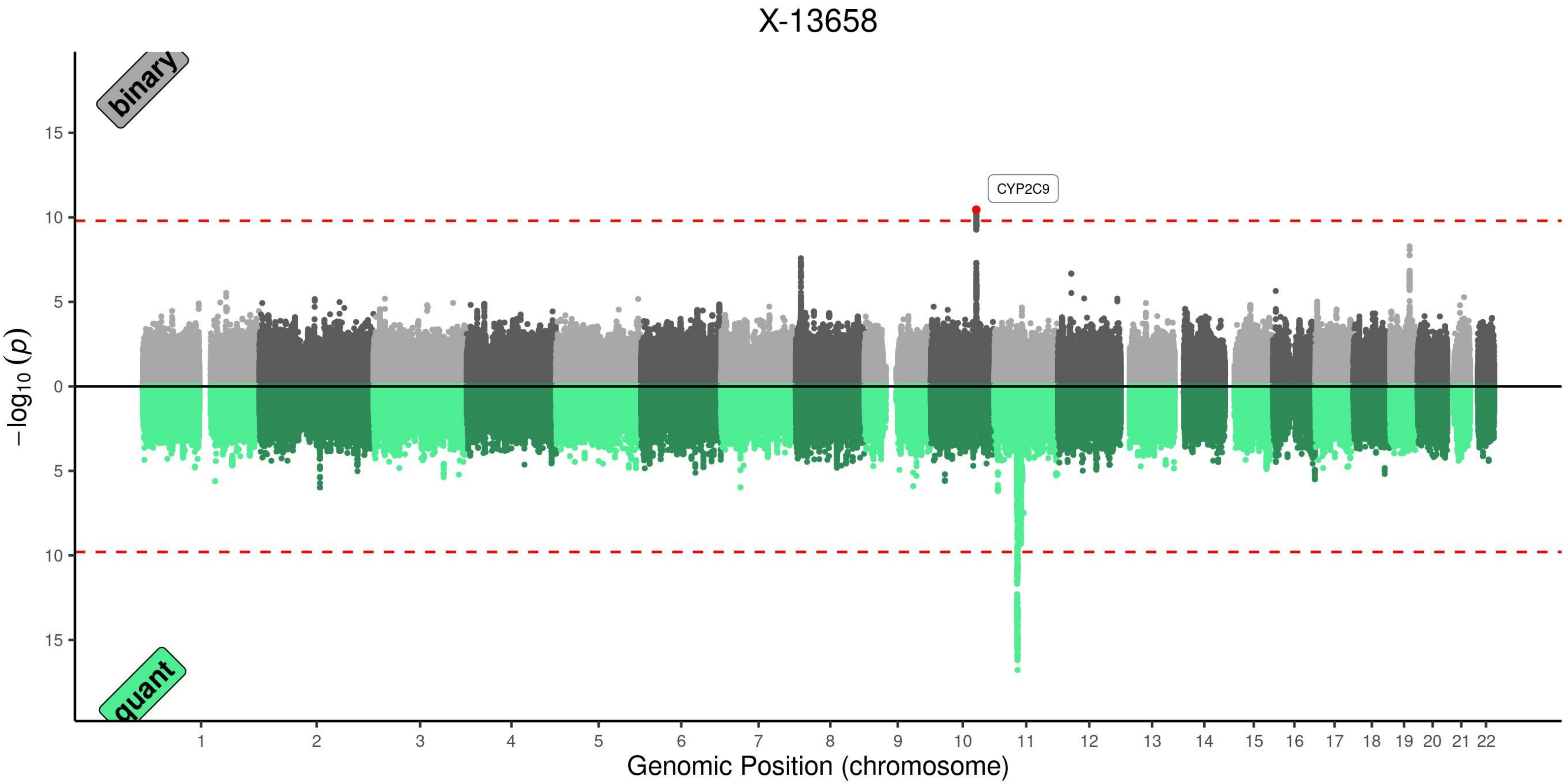

X-13658
binary
quant
CYP2C9
15
10
5
0
5
10
15
−log10 (p)
1
2
3
4
5
6
7
8
9
10
11
12
13
14
15
16
17
18
19
20
21
22
Genomic Position (chromosome)

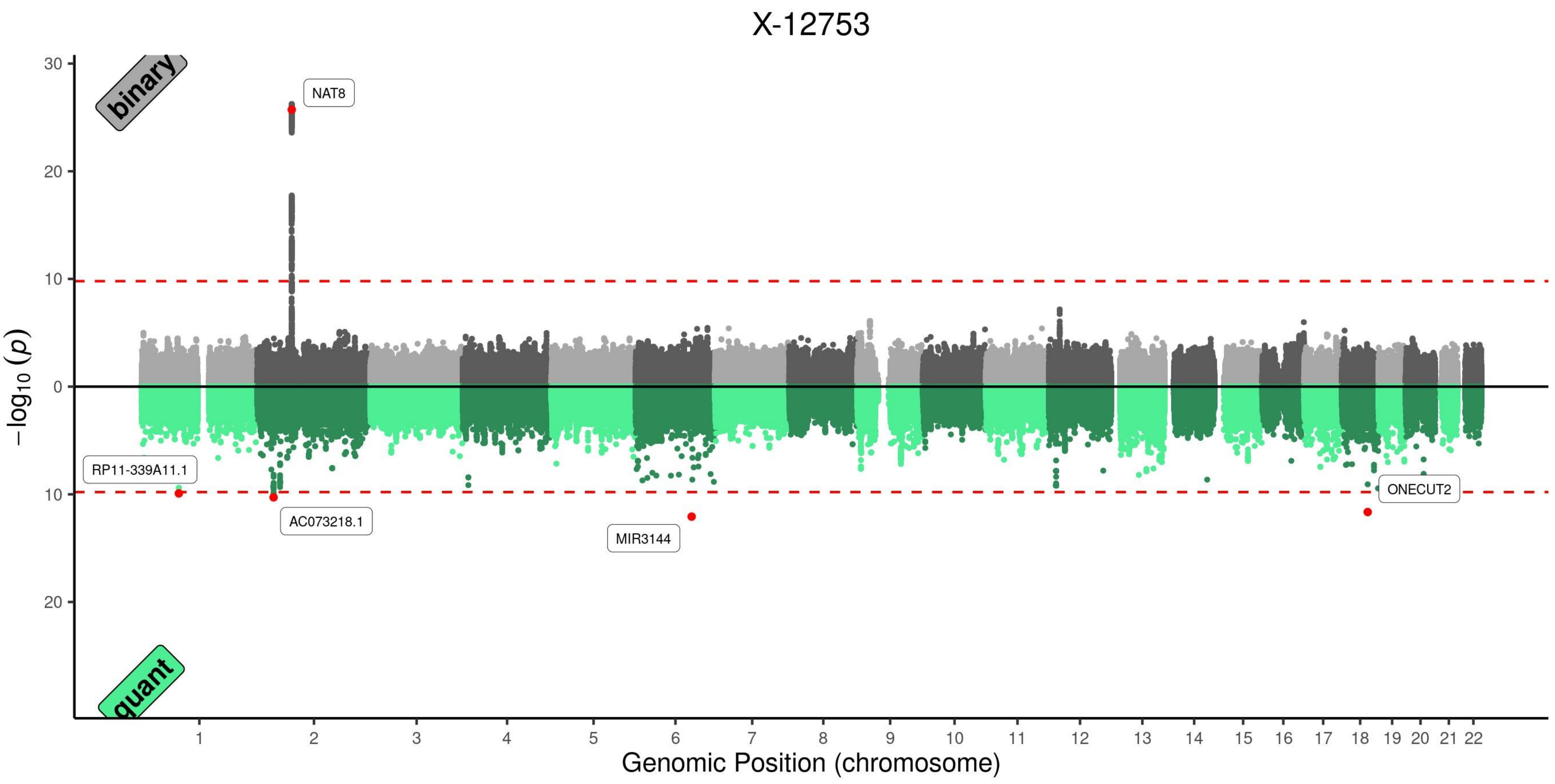

X-12753
binary
quant
NAT8
RP11-339A11.1
AC073218.1
MIR3144
ONECUT2
30
20
10
0
10
20
−log10 (p)
1
2
3
4
5
6
7
8
9
10
11
12
13
14
15
16
17
18
19
20
21
22
Genomic Position (chromosome)

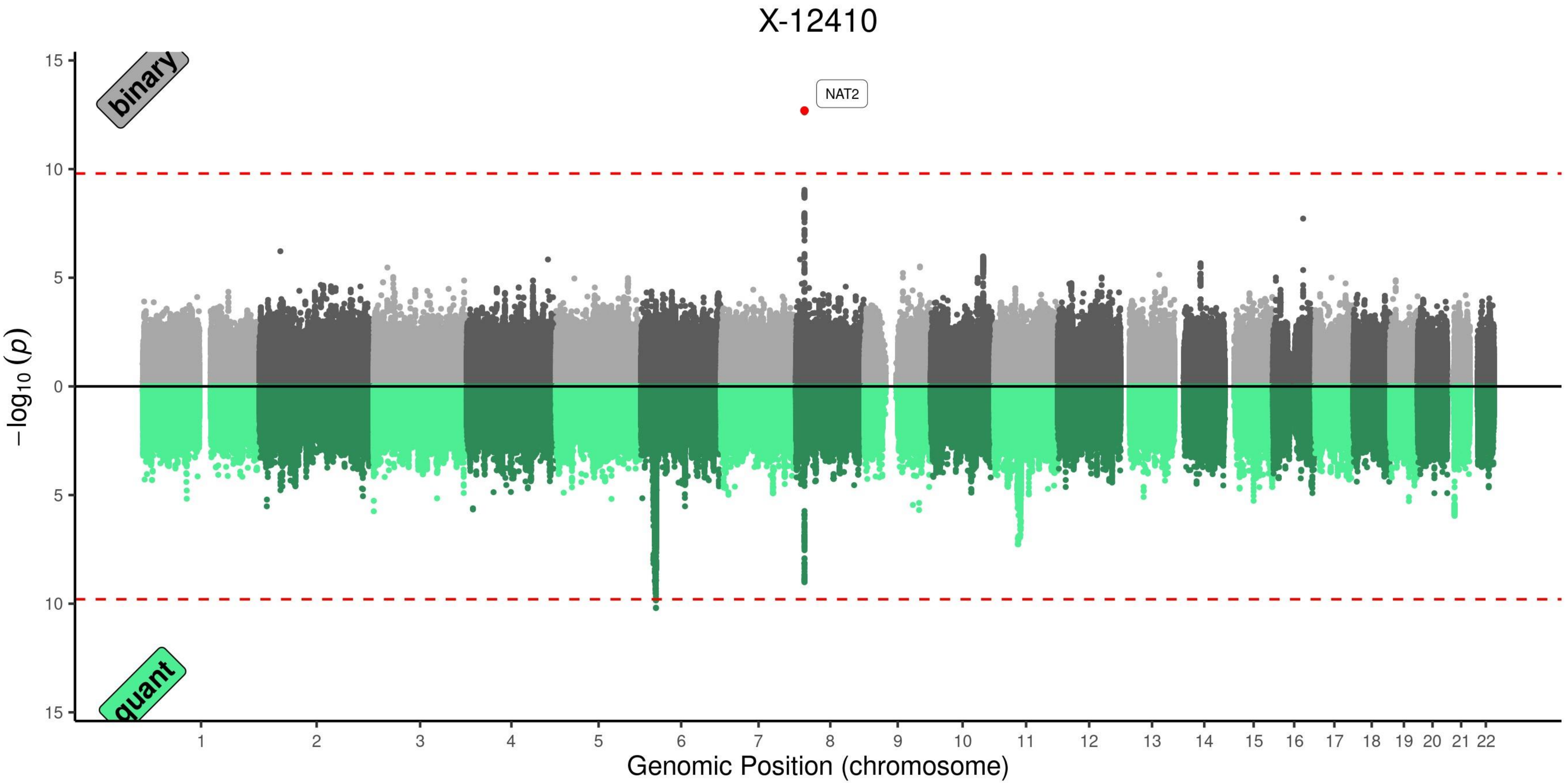
X-12410
binary
quant
NAT2
−log10 (p)
15
10
5
0
5
10
15
1
2
3
4
5
6
7
8
9
10
11
12
13
14
15
16
17
18
19
20
21
22
Genomic Position (chromosome)

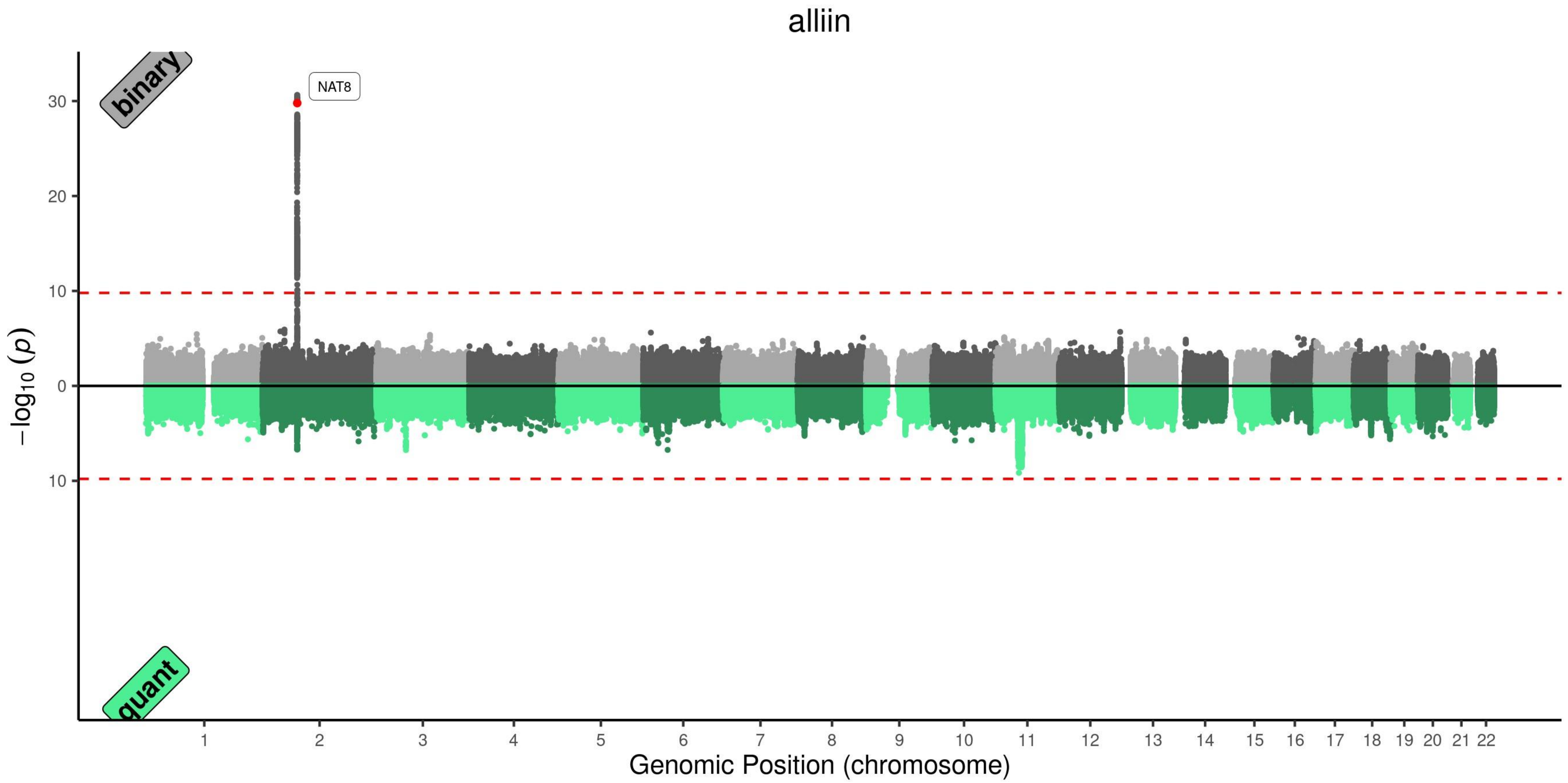

alliin
binary
quant
NAT8
30
20
10
0
10
−log10 (p)
1
2
3
4
5
6
7
8
9
10
11
12
13
14
15
16
17
18
19
20
21
22
Genomic Position (chromosome)

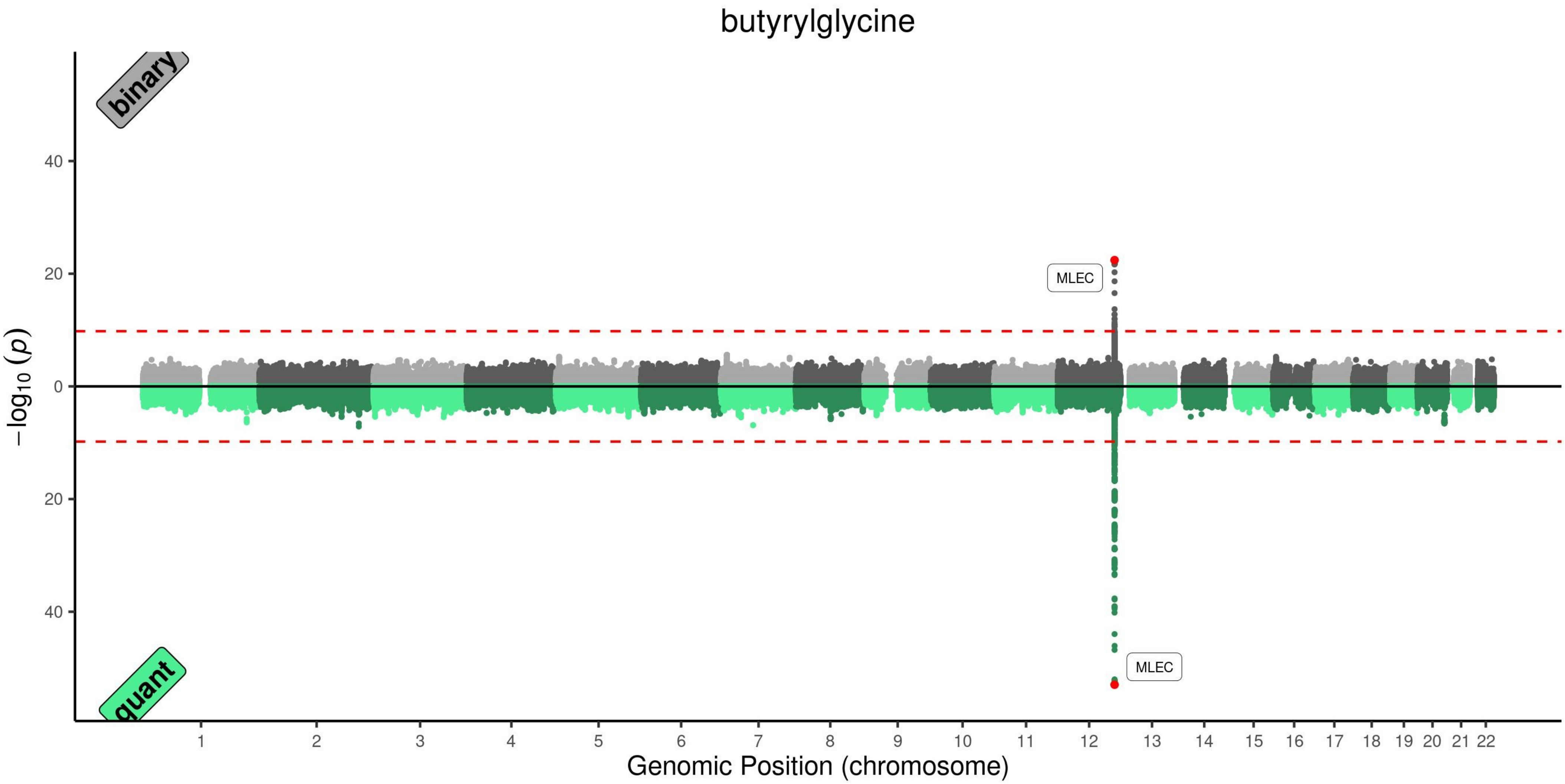
butyrylglycine
binary
quant
MLEC
MLEC
40
20
0
20
40
−log10 (p)
1
2
3
4
5
6
7
8
9
10
11
12
13
14
15
16
17
18
19
20
21
22
Genomic Position (chromosome)

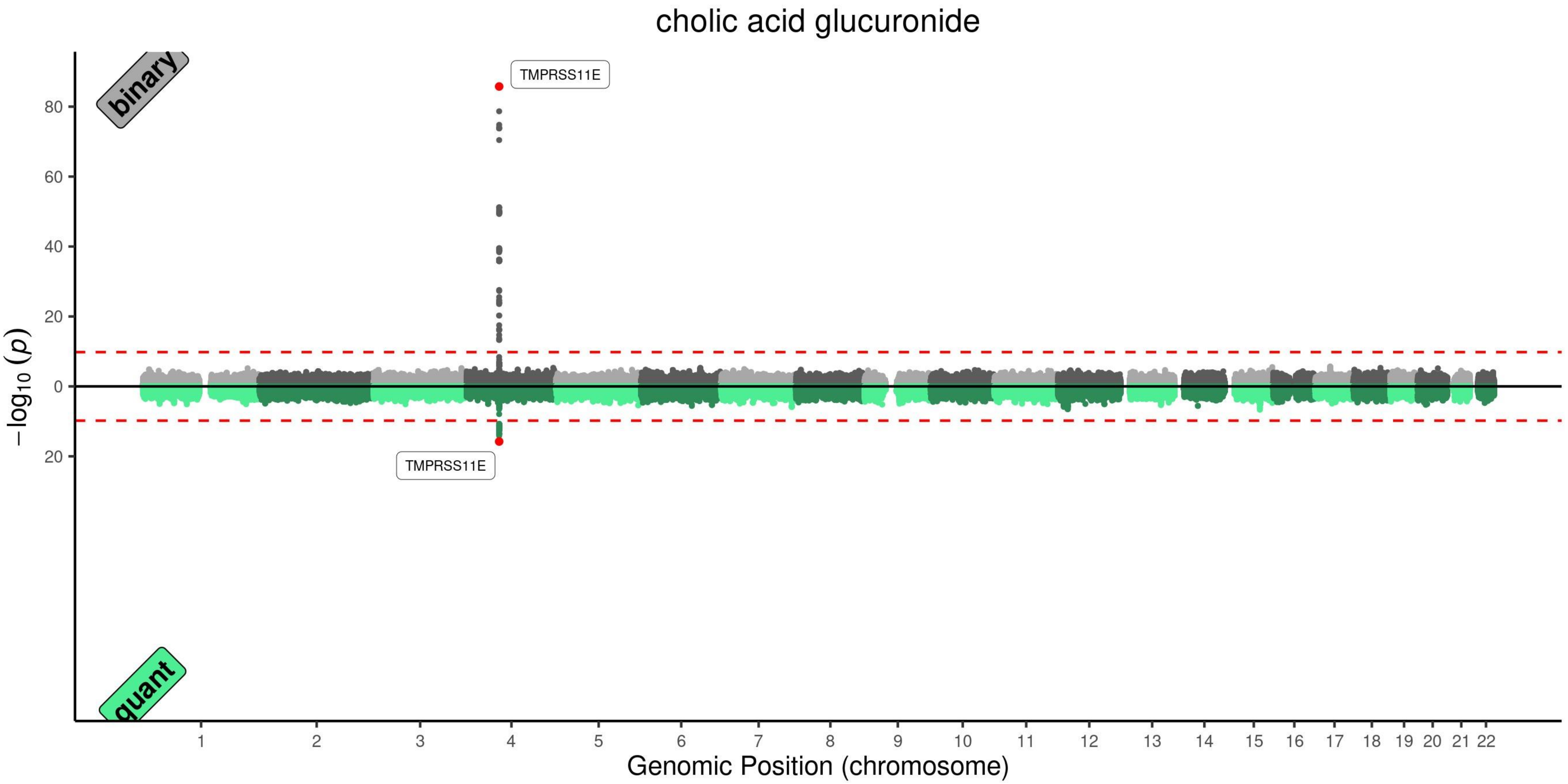

cholic acid glucuronide
binary
quant
TMPRSS11E
TMPRSS11E
80
60
40
20
0
20
$-\log_{10}(p)$
1 2 3 4 5 6 7 8 9 10 11 12 13 14 15 16 17 18 19 20 21 22
Genomic Position (chromosome)

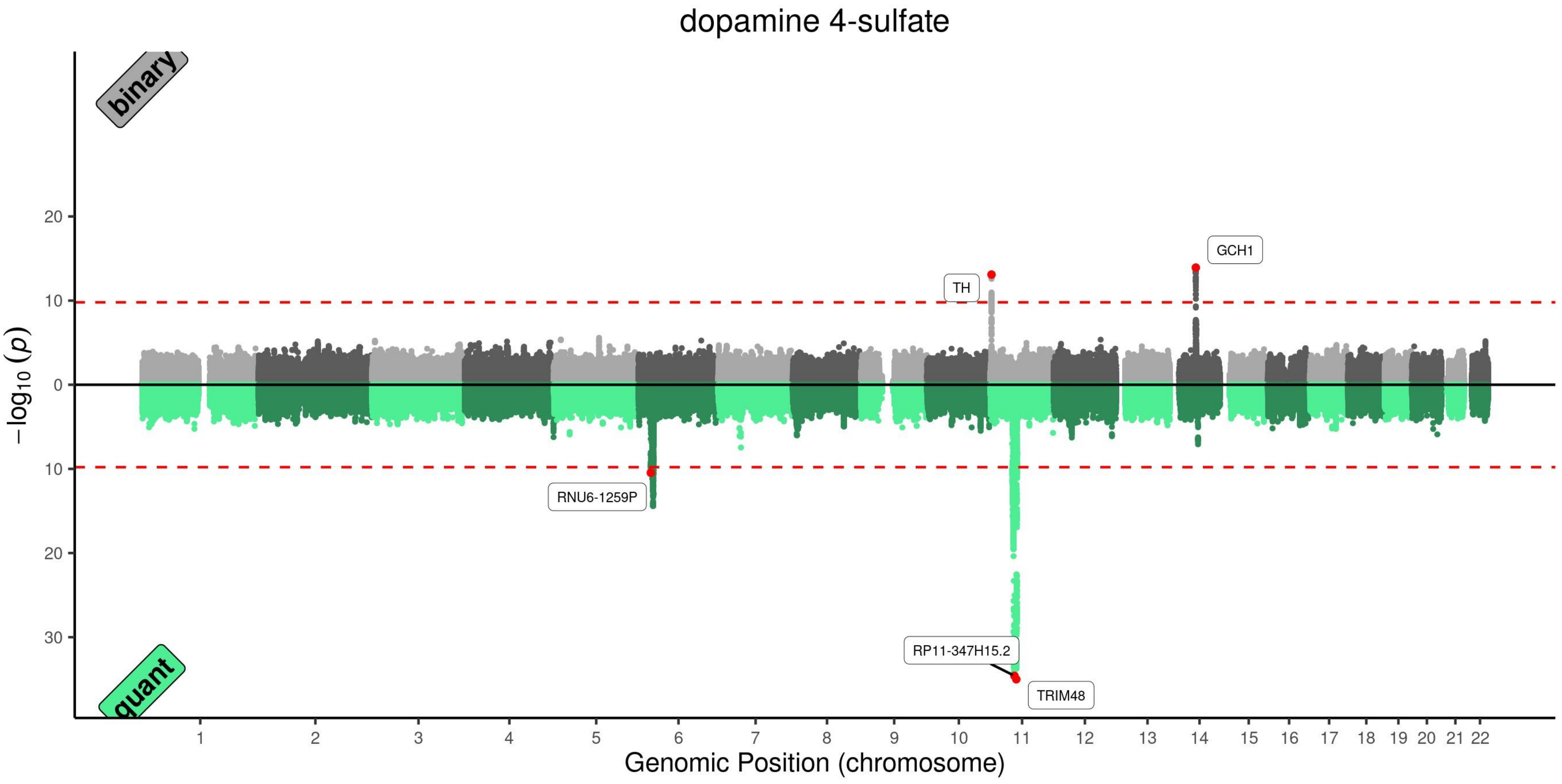

dopamine 4-sulfate
binary
quant
−log10(p)
20
10
0
10
20
30
TH
GCH1
RNU6-1259P
RP11-347H15.2
TRIM48
1
2
3
4
5
6
7
8
9
10
11
12
13
14
15
16
17
18
19
20
21
22
Genomic Position (chromosome)

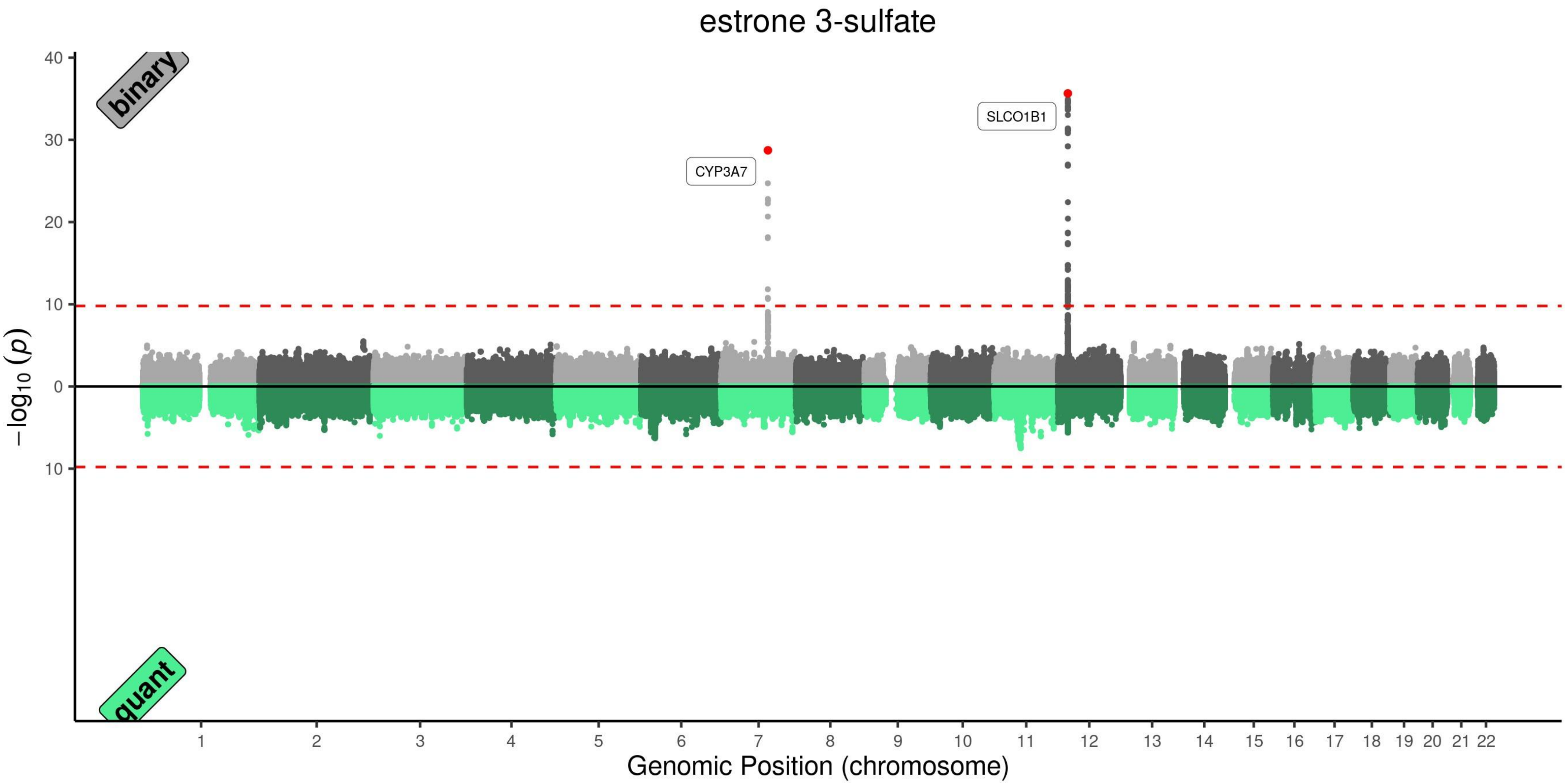

estrone 3-sulfate
binary
quant
CYP3A7
SLCO1B1
40
30
20
10
0
10
$-\log_{10}(p)$
1
2
3
4
5
6
7
8
9
10
11
12
13
14
15
16
17
18
19
20
21
22
Genomic Position (chromosome)

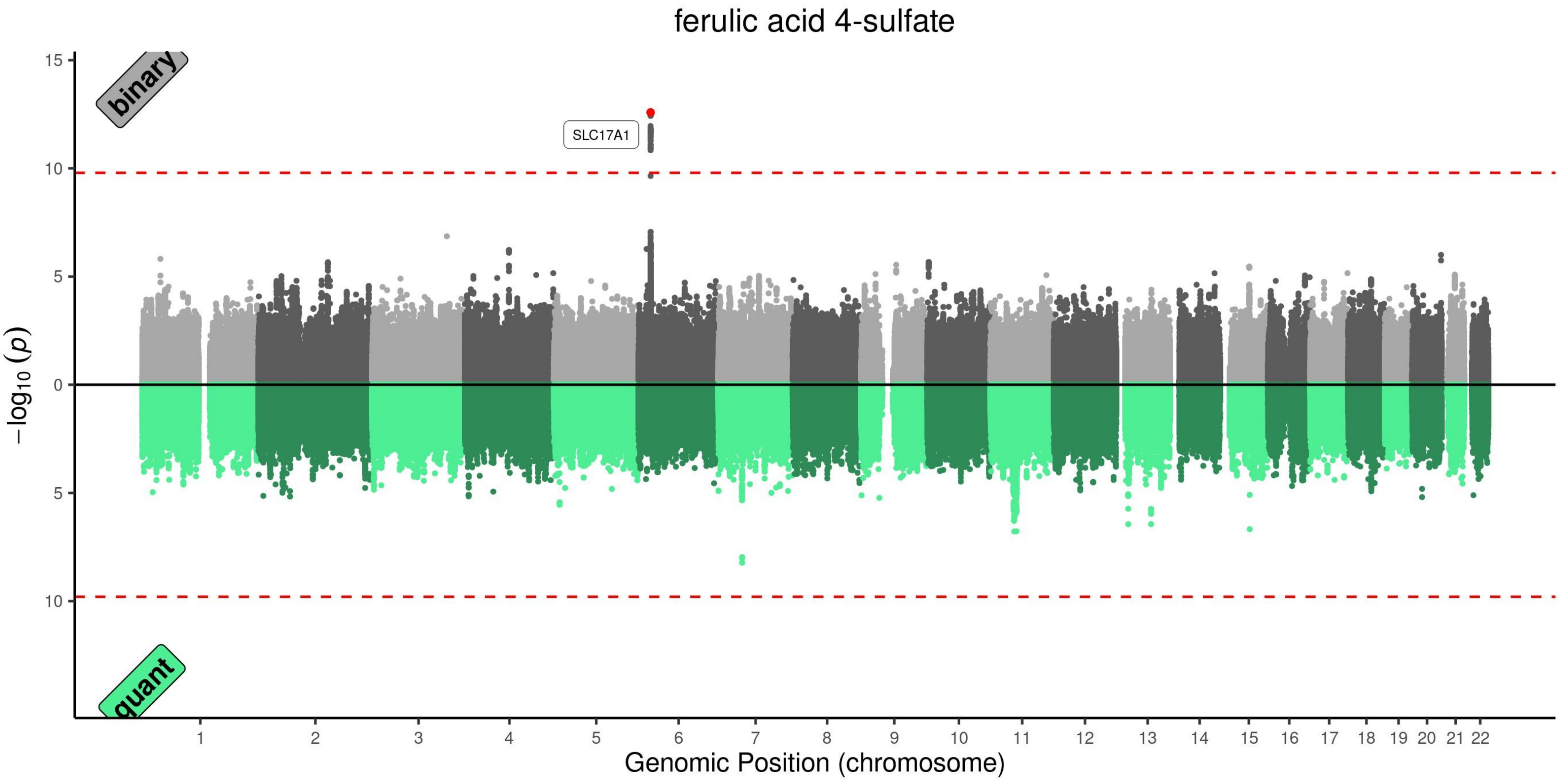

ferulic acid 4-sulfate
binary
quant
SLC17A1
−log10(p)
Genomic Position (chromosome)
15
10
5
0
5
10
1
2
3
4
5
6
7
8
9
10
11
12
13
14
15
16
17
18
19
20
21
22

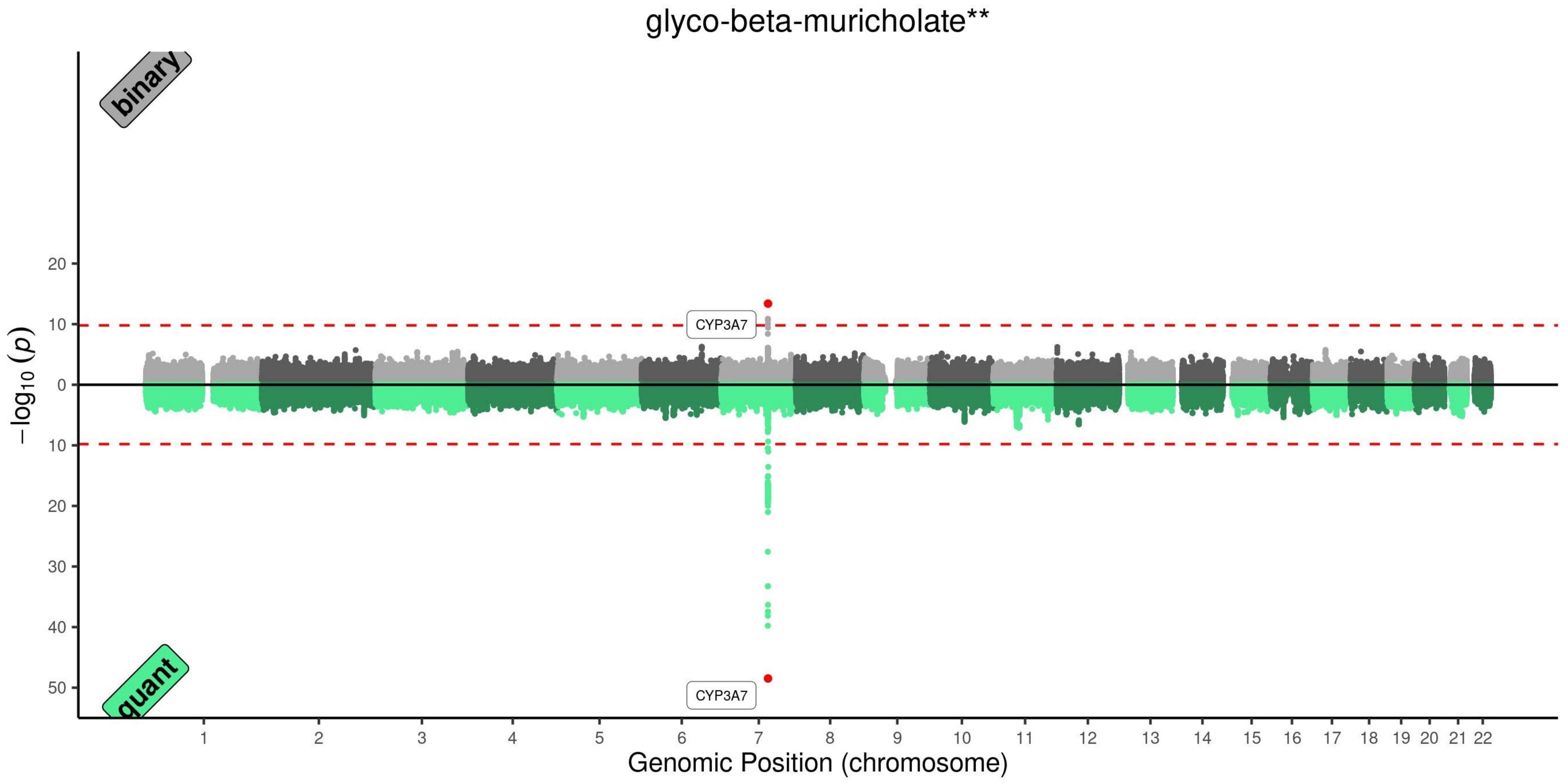
glyco-beta-muricholate**
binary
quant
CYP3A7
CYP3A7
−log10 (p)
20
10
0
10
20
30
40
50
1 2 3 4 5 6 7 8 9 10 11 12 13 14 15 16 17 18 19 20 21 22
Genomic Position (chromosome)

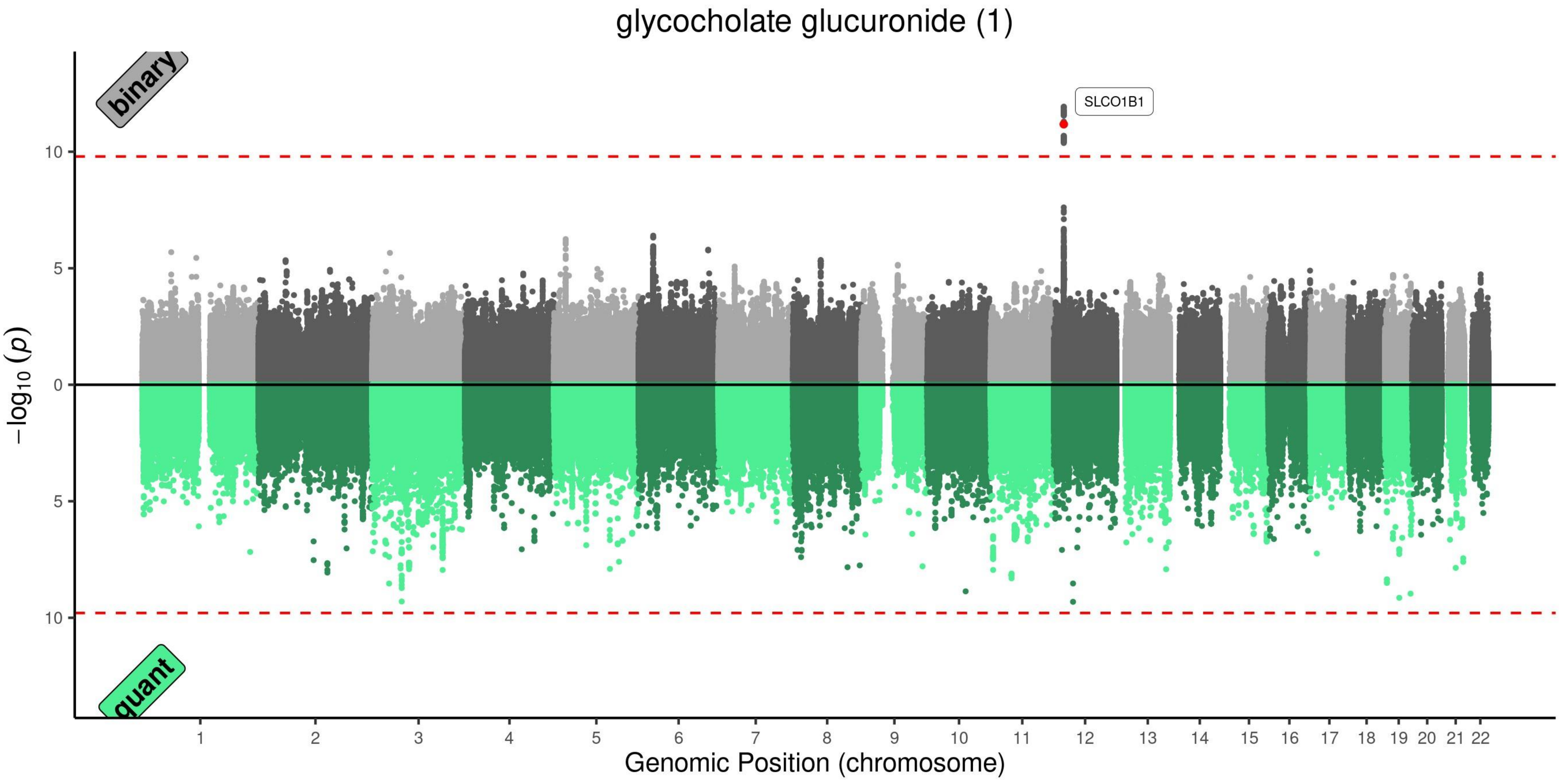

glycocholate glucuronide (1)
binary
quant
SLCO1B1
10
5
0
5
10
−log10 (p)
1
2
3
4
5
6
7
8
9
10
11
12
13
14
15
16
17
18
19
20
21
22
Genomic Position (chromosome)

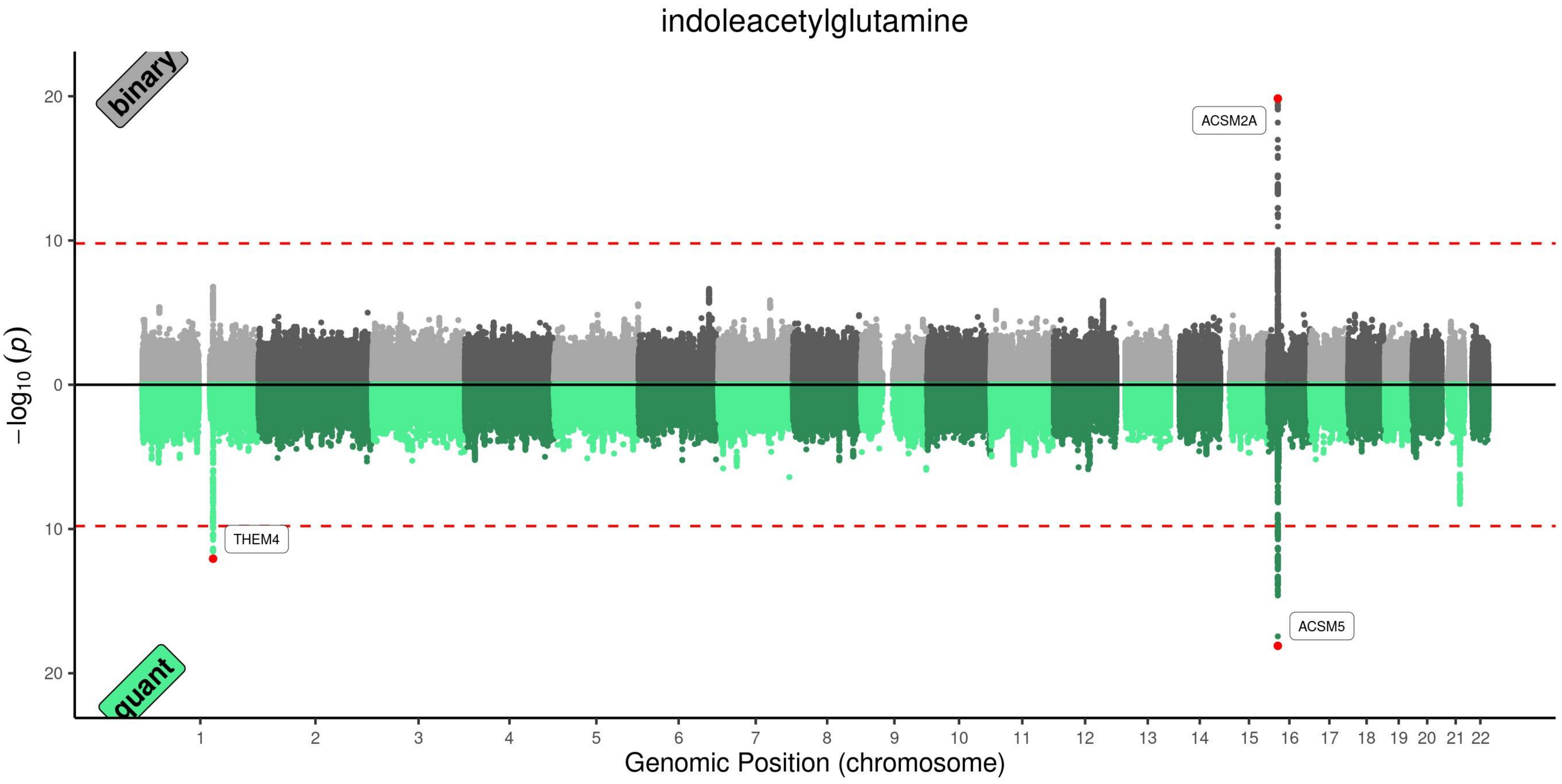

indoleacetylglutamine
binary
quant
ACSM2A
THEM4
ACSM5
20
10
0
10
20
−log10 (p)
1
2
3
4
5
6
7
8
9
10
11
12
13
14
15
16
17
18
19
20
21
22
Genomic Position (chromosome)

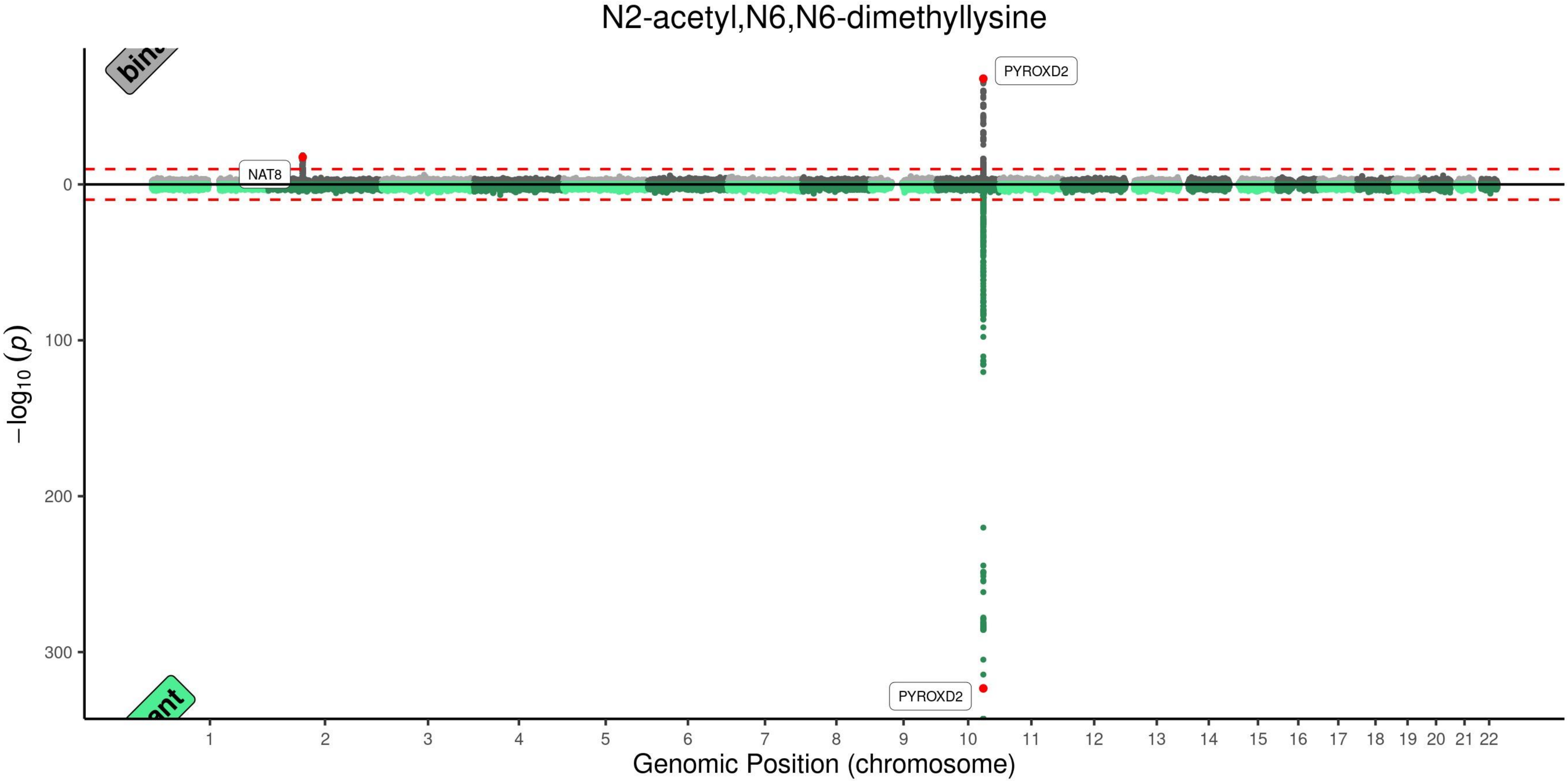

N2-acetyl,N6,N6-dimethyllysine
PYROXD2
NAT8
PYROXD2
0
100
200
300
−log10 (p)
1
2
3
4
5
6
7
8
9
10
11
12
13
14
15
16
17
18
19
20
21
22
Genomic Position (chromosome)

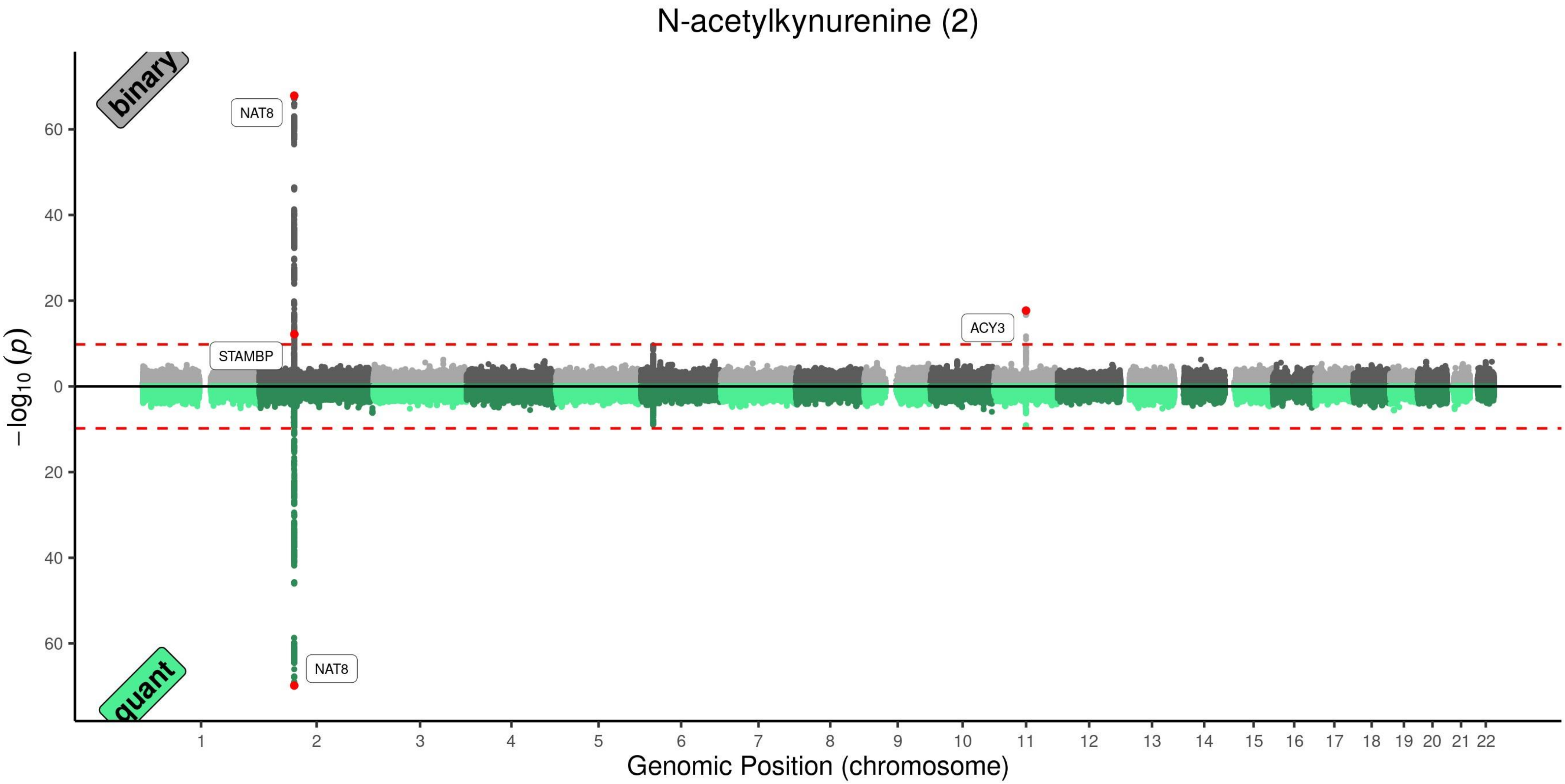

N-acetylkynurenine (2)
binary
quant
NAT8
STAMBP
ACY3
NAT8
60
40
20
0
20
40
60
−log10 (p)
1
2
3
4
5
6
7
8
9
10
11
12
13
14
15
16
17
18
19
20
21
22
Genomic Position (chromosome)

pregnanolone/allopregnanolone sulfate

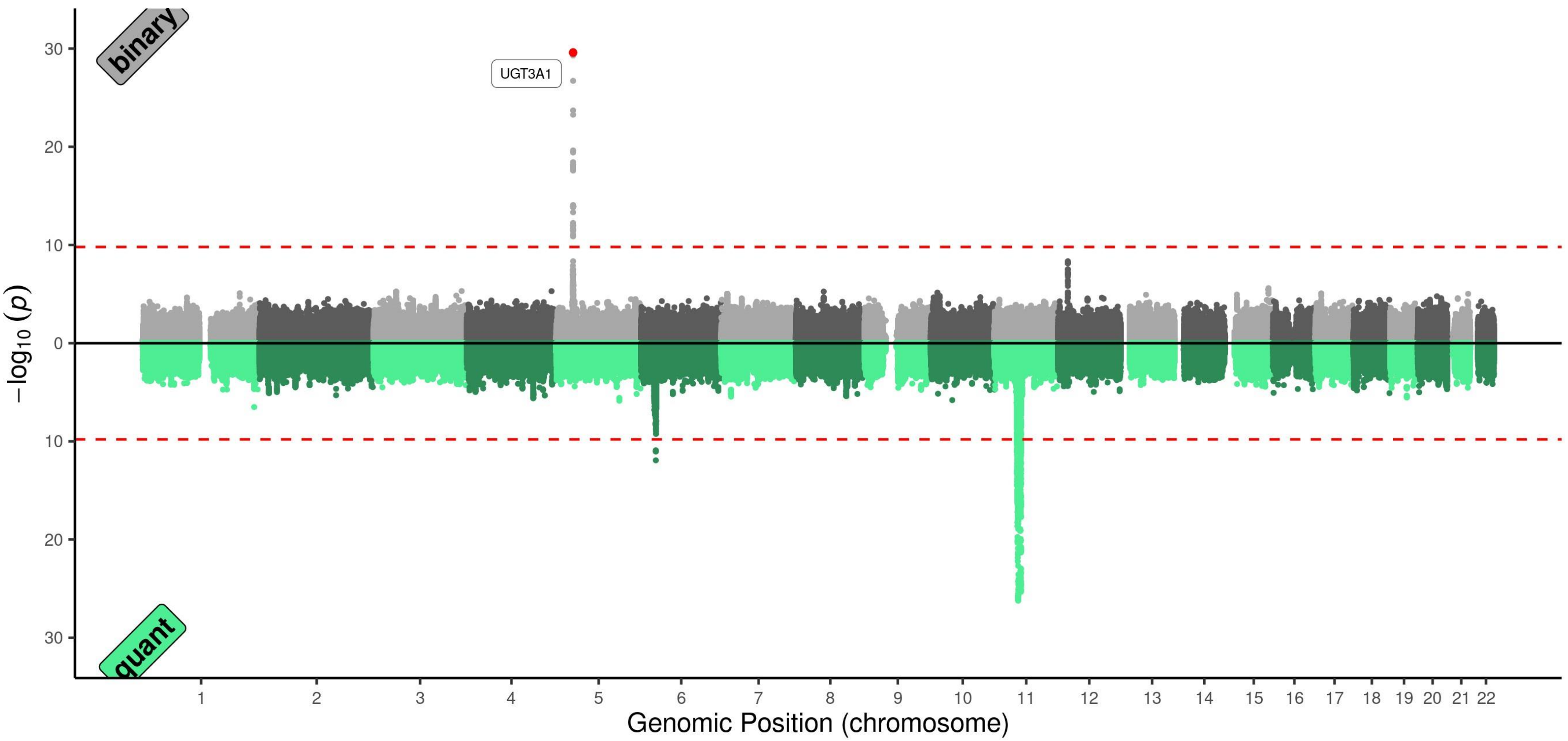

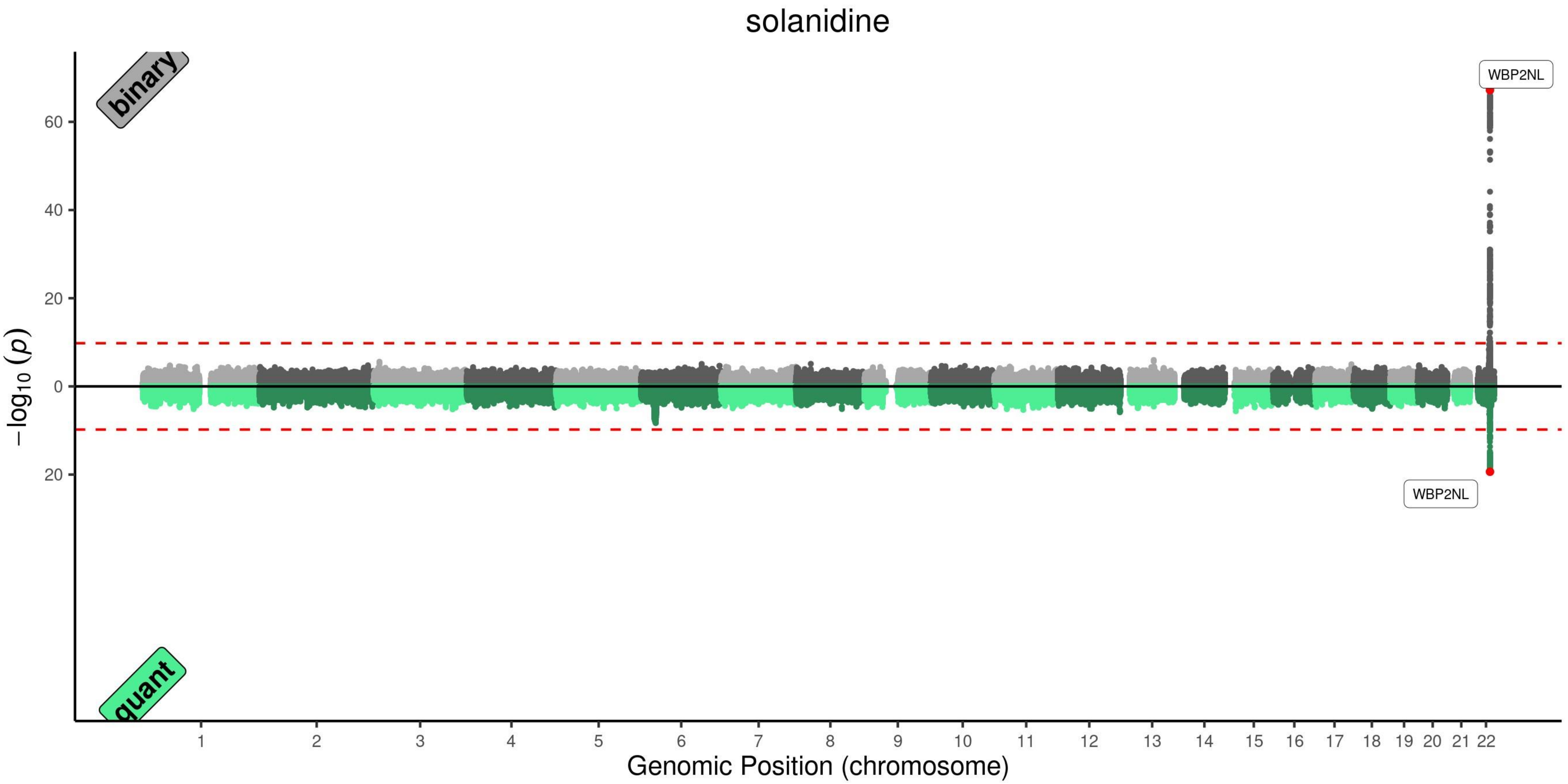
solanidine
binary
quant
WBP2NL
WBP2NL
60
40
20
0
20
$-\log_{10}(p)$
1
2
3
4
5
6
7
8
9
10
11
12
13
14
15
16
17
18
19
20
21
22
Genomic Position (chromosome)

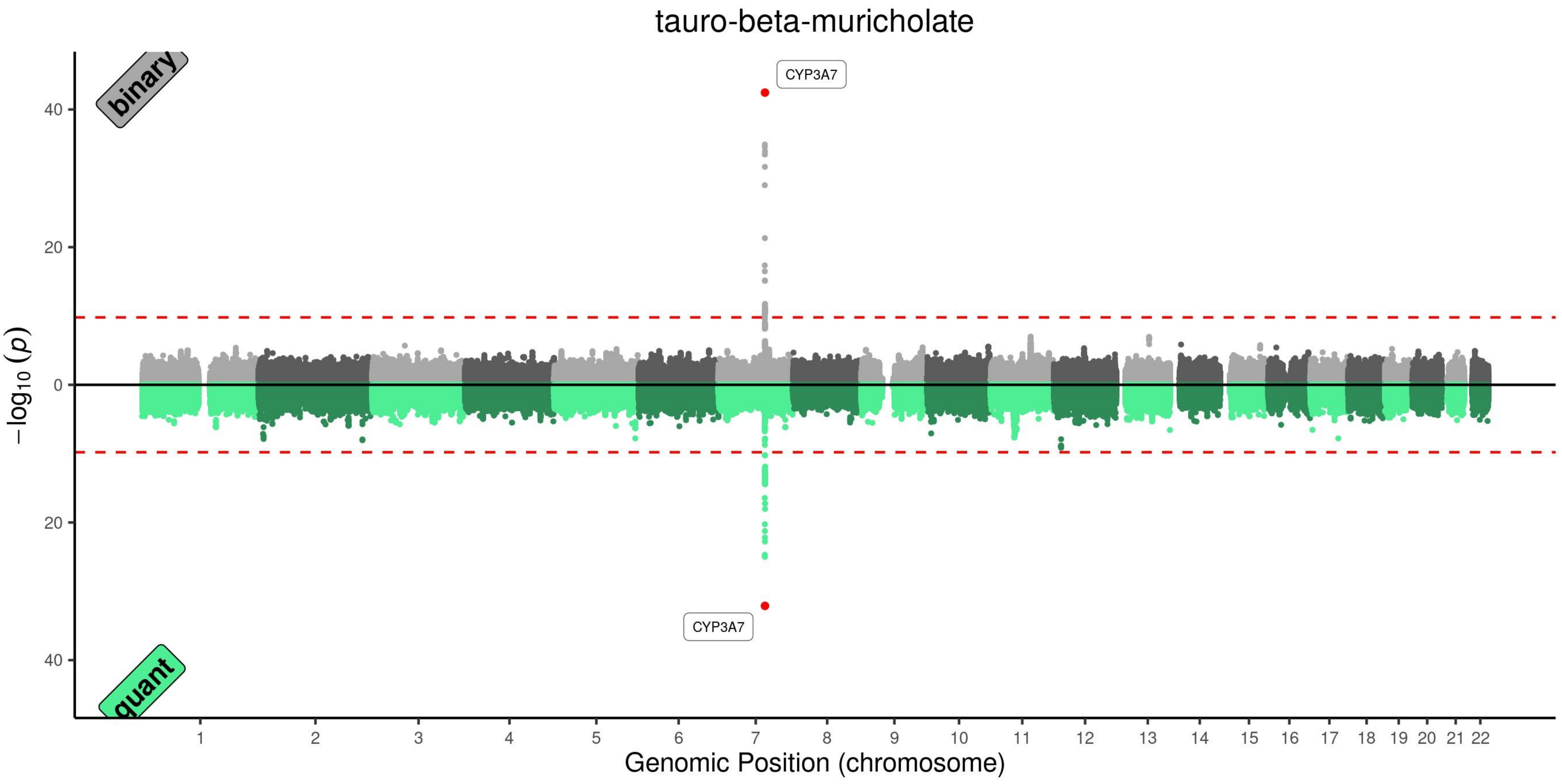

tauro-beta-muricholate
binary
quant
CYP3A7
CYP3A7
$-\log_{10}(p)$
40
20
0
20
40
1
2
3
4
5
6
7
8
9
10
11
12
13
14
15
16
17
18
19
20
21
22
Genomic Position (chromosome)

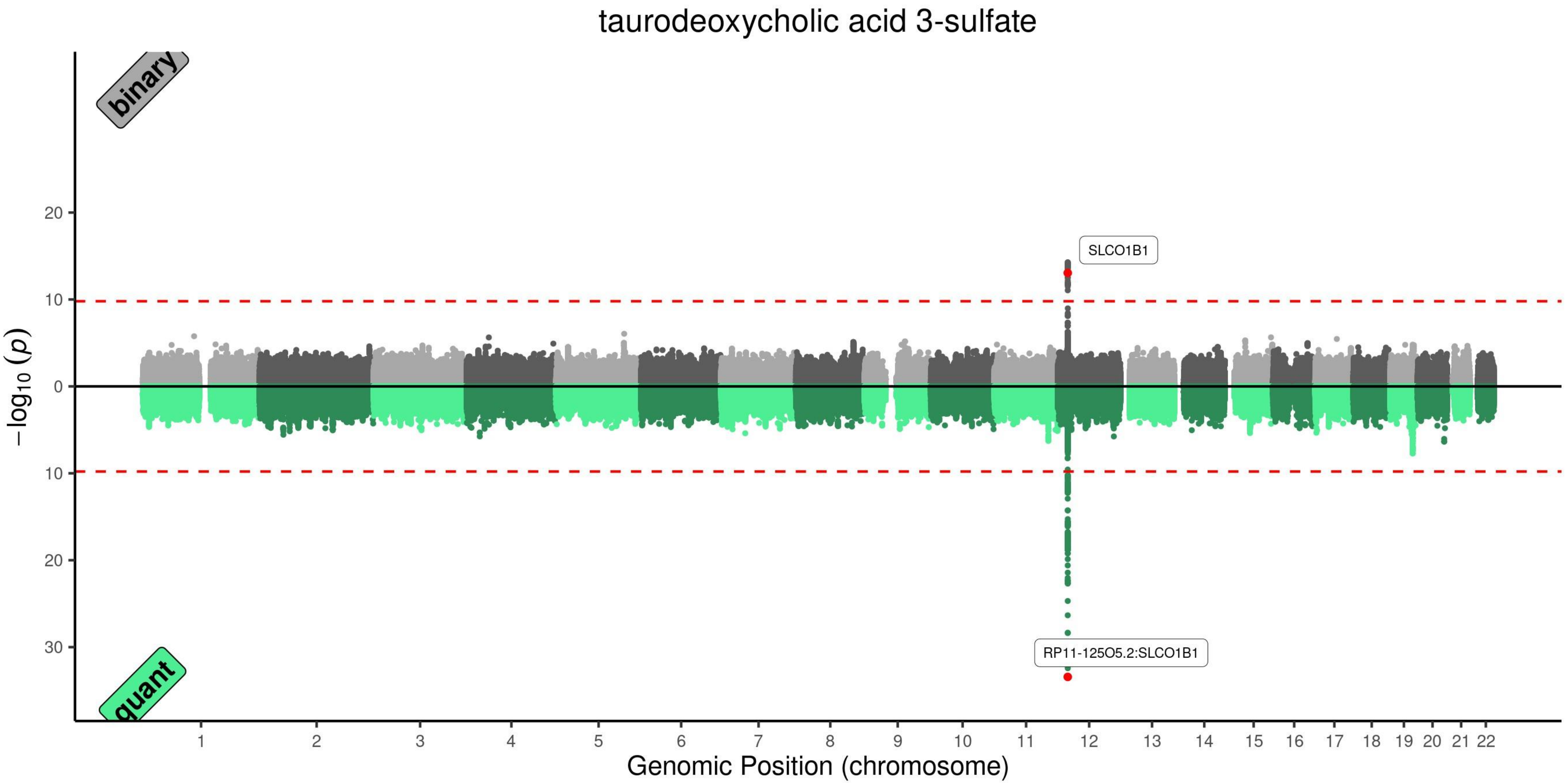
taurodeoxycholic acid 3-sulfate
binary
quant
SLCO1B1
RP11-125O5.2:SLCO1B1
−log10 (p)
20
10
0
10
20
30
1
2
3
4
5
6
7
8
9
10
11
12
13
14
15
16
17
18
19
20
21
22
Genomic Position (chromosome)

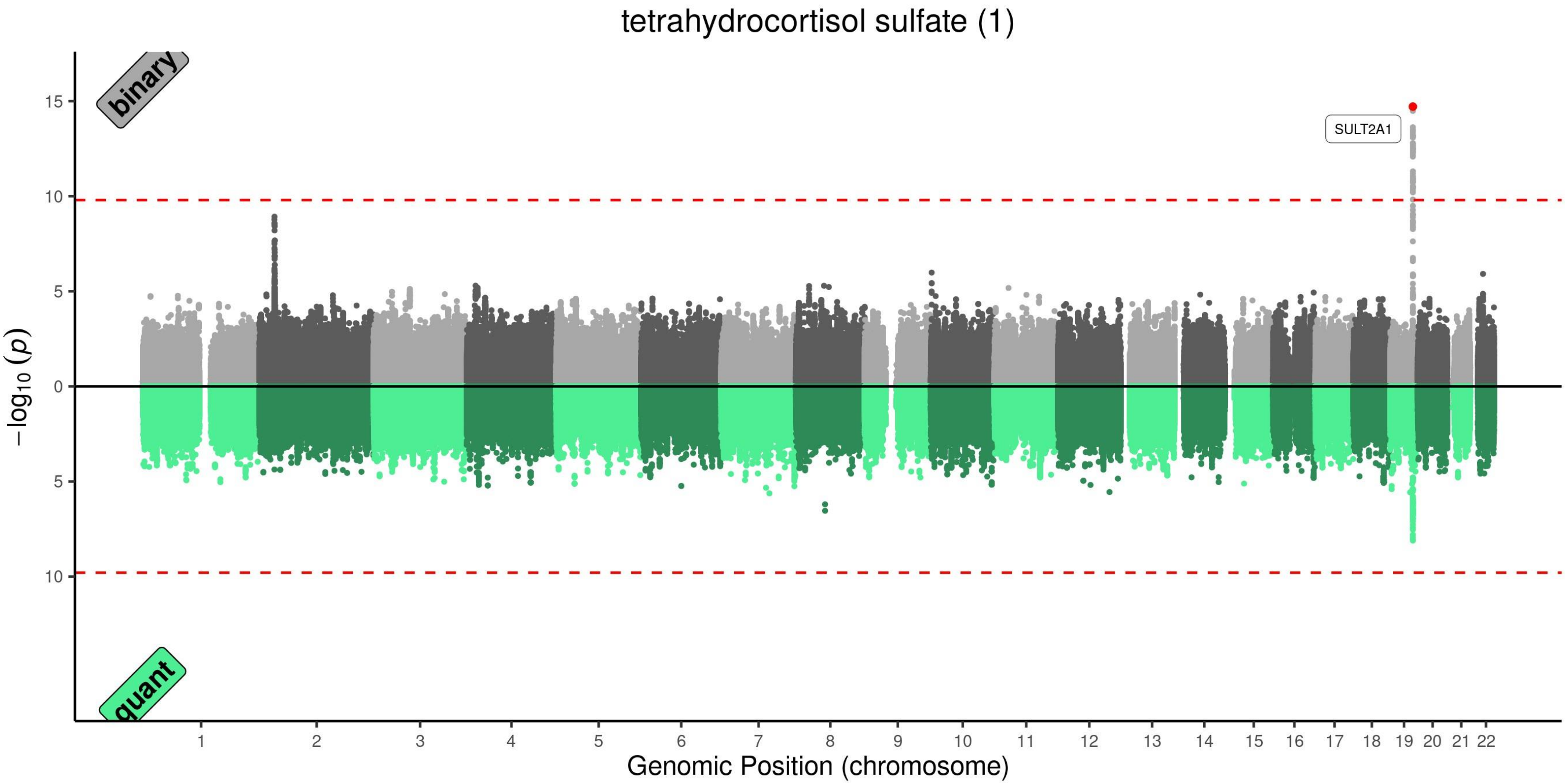

tetrahydrocortisol sulfate (1)
binary
quant
SULT2A1
−log10 (p)
15
10
5
0
5
10
1
2
3
4
5
6
7
8
9
10
11
12
13
14
15
16
17
18
19
20
21
22
Genomic Position (chromosome)

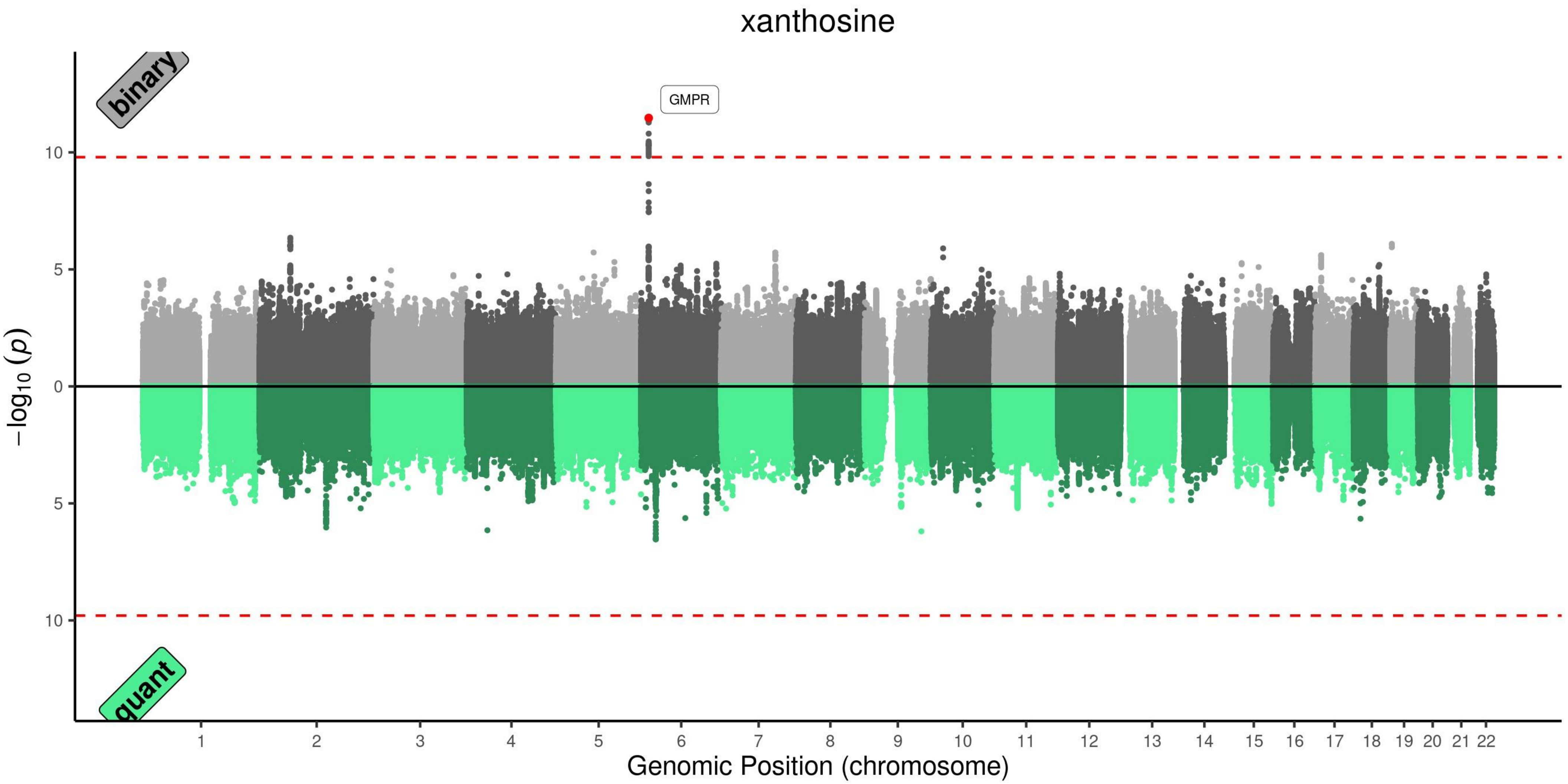
xanthosine
binary
quant
GMPR
−log10(p)
10
5
0
5
10
1
2
3
4
5
6
7
8
9
10
11
12
13
14
15
16
17
18
19
20
21
22
Genomic Position (chromosome)

Fig. S2. Correlation between the GWAS meta-analysis independent SNP (r^2<0.6)-metabolite effect sizes from the Rhineland Study and the NEO Study

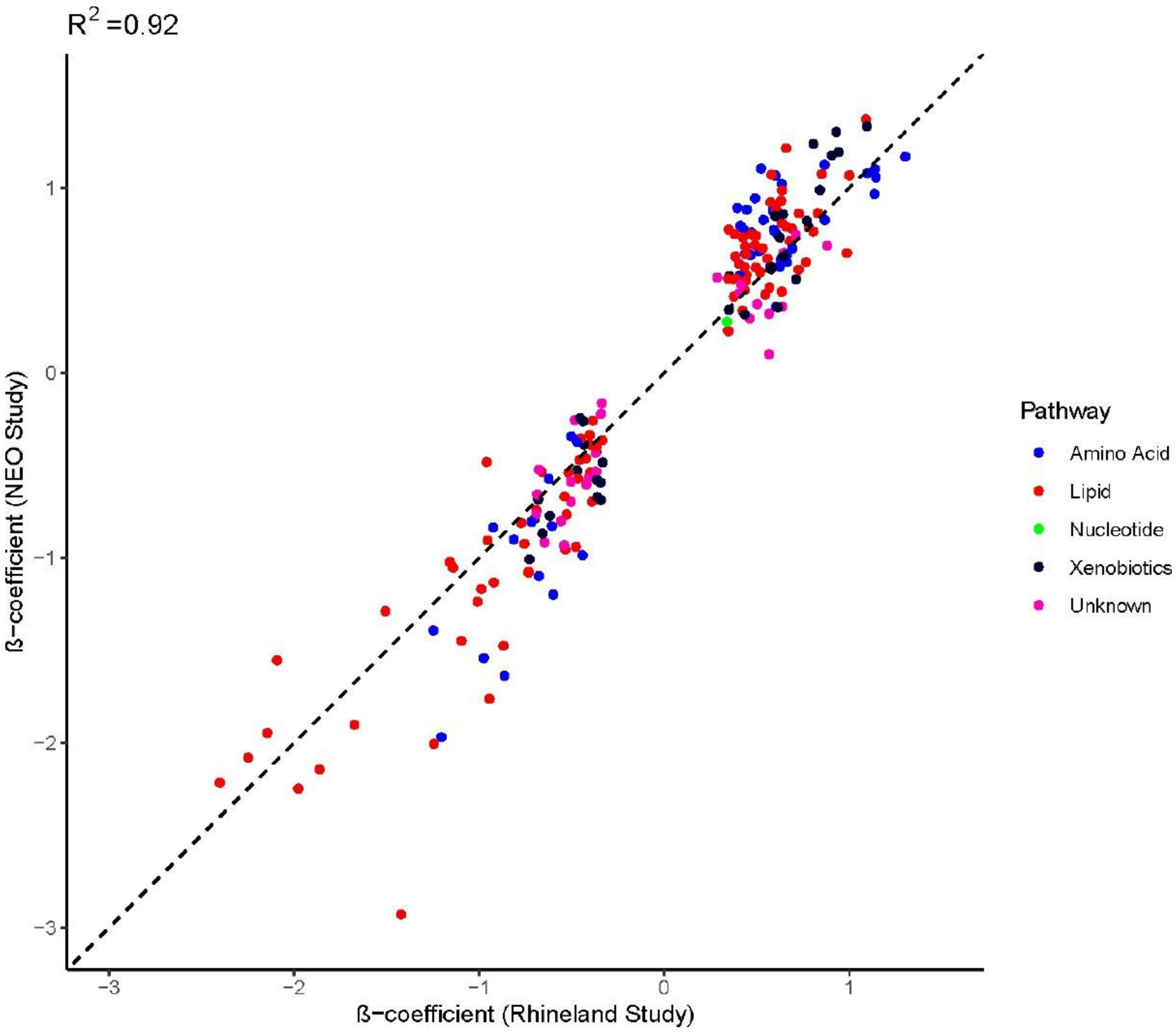

Fig. S3. Correlation and visualization of missingness of 3-decenoylcarnitine and dopamine 4-sulphate in relation to carnitine and dopamine 3-sulphate in the NEO study

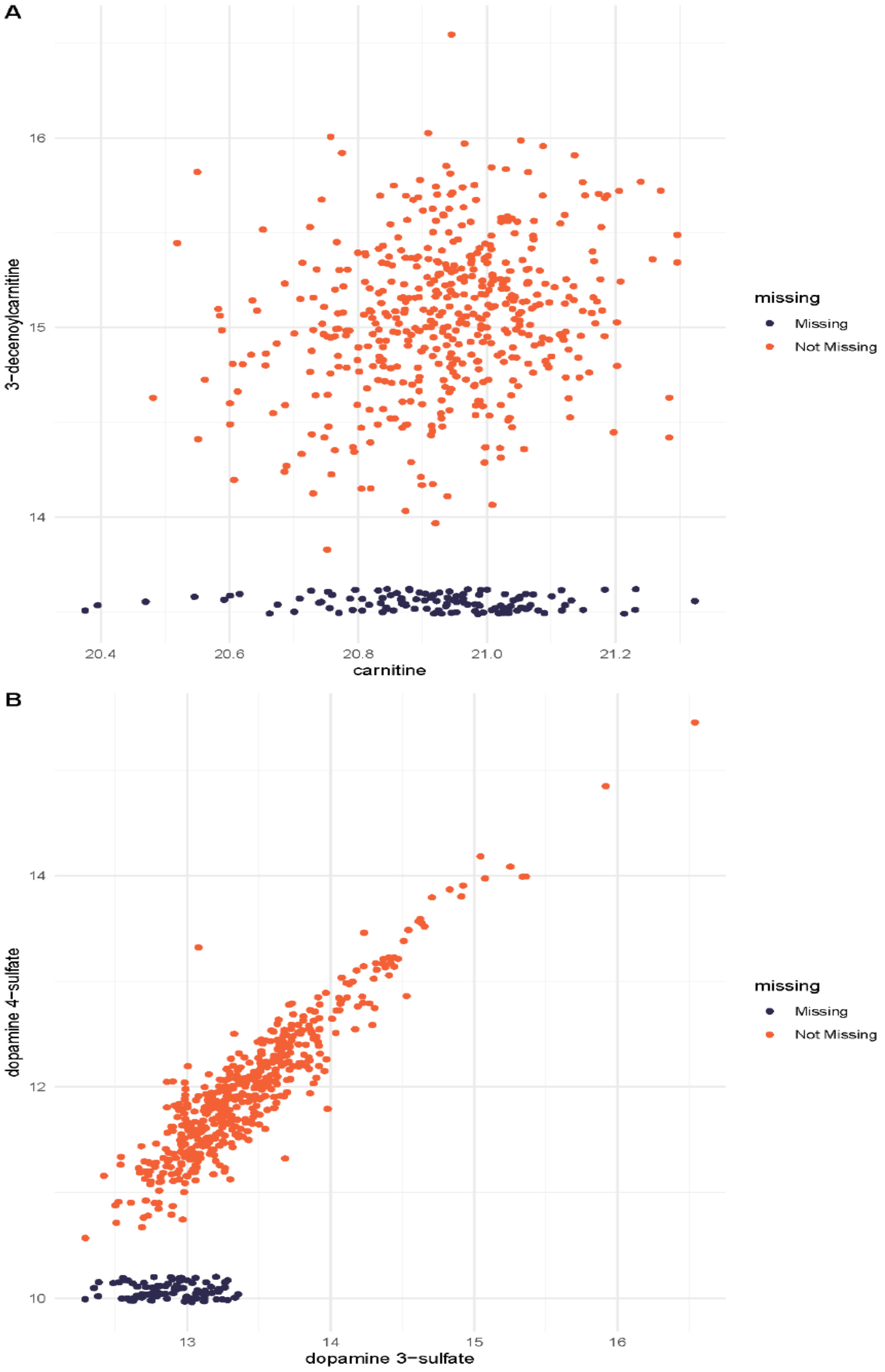

Fig. S4. Correlation and visualization of missingness of 3-decenoylcarnitine and dopamine 4-sulphate in relation to carnitine and dopamine 3-sulphate in the Rhineland Study

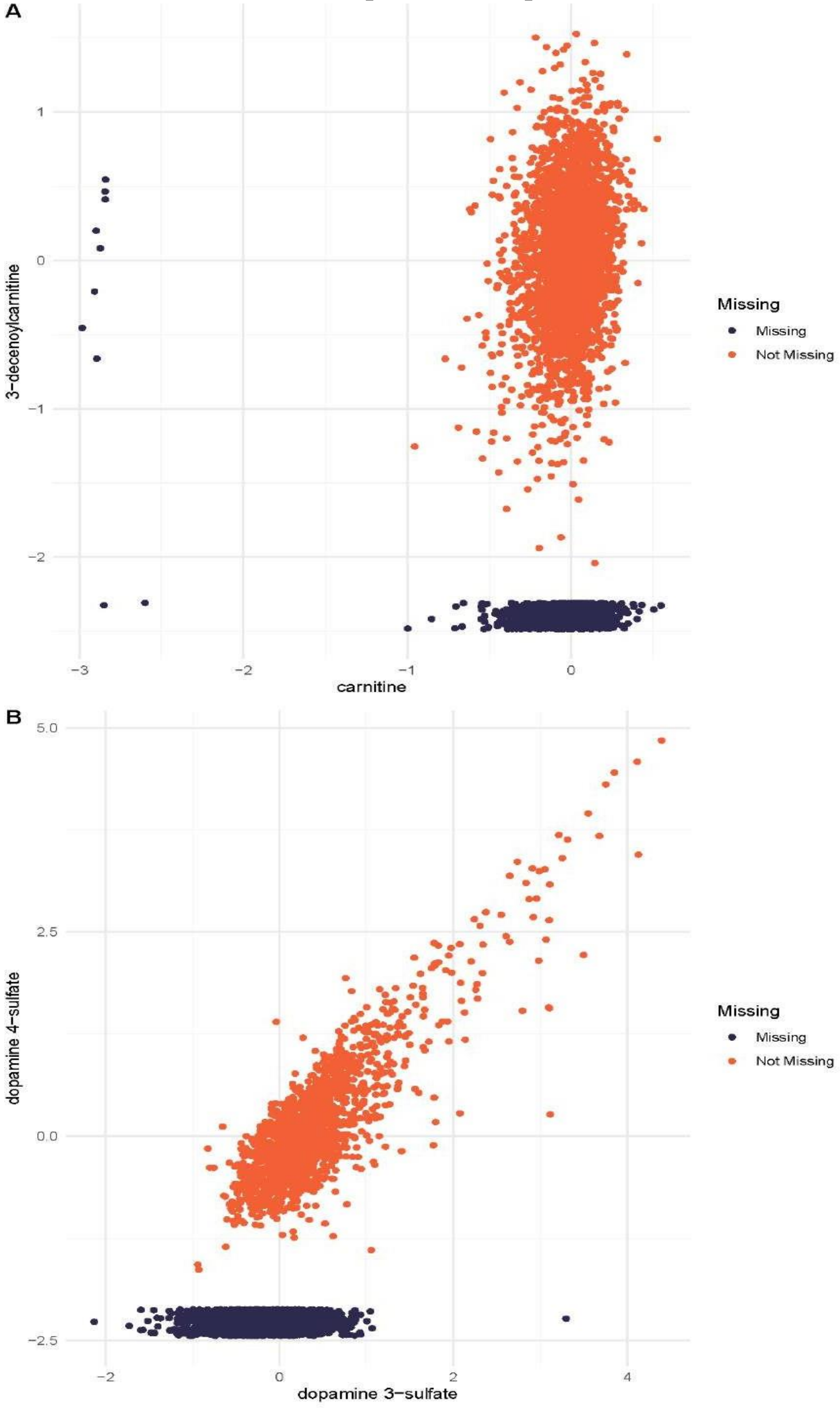

Supplement: Supplementary file 1 — Additional file 1: Figs. S1-S4. Supplementary figures showing quantitative GWAS sensitivity analyses, correlation of SNP effect estimates between NEO and Rhineland study cohorts, and visualization of missingness of 3-decenoylcarnitine and dopamine 4-sulphate in relation to carnitine and dopamine 3-sulphate in NEO and Rhineland study cohorts [file 13059_2026_4132_MOESM1_ESM.pdf]
